# Multi-pathogen and combination vaccines for biodefense pathogens

**DOI:** 10.3389/fimmu.2026.1945700

**Published:** 2026-10-16

**Authors:** Elizabeth C. Clarke, Ria Q. Kidner, Makaela M. Montoya, Kiersten D. Lenz, Jessica Z. Kubicek-Sutherland, Steven B. Bradfute

**Affiliations:** 1 Molecular Genetics and Microbiology, University of New Mexico Health Sciences Center, Albuquerque, NM, United States; 2 Physical Chemistry and Spectroscopy, Chemistry Division, Los Alamos National Laboratory, Los Alamos, NM, United States; 3 Center for Global Health, Department of Internal Medicine, University of New Mexico Health Sciences Center, Albuquerque, NM, United States

**Keywords:** biodefense, biothreat, combination vaccines, multi-pathogen, multivalent, vaccine interference, vaccines

## Abstract

Multi-pathogen and combination vaccines are vaccines that deliver antigens from two or more pathogens in a single shot to protect against multiple diseases. This can include several individual vaccines formulated together to be delivered as a single shot as well as multivalent vaccines, where a single vaccine expresses or contains multiple antigens. Multi-pathogen vaccines present an obvious solution for rapidly protecting a population against multiple biological threat agents simultaneously. They also have additional cost and logistical advantages over delivering separate vaccines. However, data from childhood combination vaccines have shown that they do not always perform as the sum of their parts, so ensuring that newly developed multi-pathogen vaccines protect as well as their individual vaccine counterparts can be challenging. Here, we focus on three core mechanisms of vaccine interference; epitope competition, resource competition and immune polarization, and discuss how multi-pathogen vaccines across different platforms (viral vectors, bacterial vectors, protein/subunit and nucleic acid) are impacted by each mechanism. We review multi-pathogen vaccines in development for pathogens and toxins that pose a biological threat, and propose an evaluation metric for assessing potential adverse combinations of vaccines. This review is intended to provide information relevant for platform-selection for developing biodefense vaccines for stockpiling and rapid deployment.

## 1 Introduction

Biological agents are a significant threat to the national population, economy, and security (1, 2). Whether intentionally engineered or naturally occurring, biological threats including viruses, bacteria, and toxins require prepared response efforts (1, 3). The need for new vaccines against biological agents has driven interest in combining antigens from various pathogens into multi-pathogen vaccine platforms (4).

Vaccines are important to overall biodefense as they serve to mitigate the impact of biological threats and to act as a deterrent (5). In the last 100 years, US troops have suffered more morbidity from infectious diseases than from combat (6). Biological threats to national security include risks to civilian populations, as demonstrated by the 2001 attacks where five people died after exposure to anthrax (7). Furthermore, since the 1990s, the threat of infectious diseases spreading in civilian populations from naturally occurring sources have been recognized as biosecurity threats (8). The pathogens and toxins that are most relevant to biodefense are defined disparately by different groups; here, we focus on pathogens listed by the U.S. Centers for Disease Control and Prevention (CDC) as Category A-C “Bioterrorism agents” (9). While the focus of this review is on biodefense applications, vaccines against biological threat pathogens are also applicable in public health. For example, the recombinant vesicular stomatitis virus expressing Ebola (Zaire) glycoprotein (rVSV-ZEBOV) vaccine used extensively in Ebola virus disease outbreaks since 2015 was developed with funding from the Canadian Safety and Security Program (under the management of Defence Research and Development Canada) and the U.S. government (10).

## 2 Biological threat agents

Biological threat agents include pathogens and toxins capable of causing severe disease, high mortality, or substantial public-health disruption, particularly when they are highly virulent, readily transmissible, difficult to treat or prevent, or amenable to aerosol dissemination. Major biodefense families include filoviruses, arenaviruses, hantaviruses, flaviviruses, and bacterial and toxin agents. Filoviruses, including Ebola virus and Marburg virus cause severe hemorrhagic fever and exhibit limited cross-protection between genera and species, complicating vaccine development. Arenaviruses such as Lassa and Machupo viruses cause severe hemorrhagic disease, while their rodent reservoirs, broad geographic distribution, and limited vaccine availability increase their risk. Hantaviruses are rodent-associated and cause either severe hantavirus cardiopulmonary syndrome or hemorrhagic fever with renal syndrome, but are potential biological threats due to their ability to transmit via aerosol. Arboviruses, including flaviviruses and alphaviruses, can cause severe hemorrhagic or neurological disease and pose risks associated with widespread vector transmission and geographic distribution. Bacterial and toxin threats- including *Bacillus anthracis*, *Yersinia pestis*, *Francisella tularensis*, and botulinum neurotoxins- are characterized by high pathogenicity and, for several agents, the potential for aerosol dissemination. Collectively, these agents present biodefense challenges due to their virulence, diverse transmission routes, limited vaccine coverage or cross-protection, and potential for rapid or widespread dissemination. The ability to vaccinate people with a single formulation to provide protection against multiple diseases offers logistical advantages over single-pathogen vaccines in terms of resources and time. Another consideration is the speed of vaccine deployment, which can be attained by rapid large-scale production or long-term stockpiling (3). It is possible that a successful multi-pathogen formulation might make stockpiling of the vaccine for emergency use more appealing, as it provides coverage for several possible outbreaks instead of just one, making these considerations particularly relevant for biological threat agents, which are high mortality/morbidity pathogens with the potential to cause wide-spread outbreaks.

## 3 Protecting against multiple threats with a single formulation

The terminology used in multi-pathogen vaccination can be confusing and inconsistent across sources, and so this review will use the definitions from Lauer et al., 2017 (11).

- Multi-pathogen vaccines – Any vaccine formulation against multiple pathogens that is delivered in a single administration, including both combination vaccines and multivalent vaccines against two or more pathogens.
- Combination vaccines – A vaccine administered as a single formulation but composed of separate individual vaccines, like DTaP (diphtheria, tetanus, and acellular pertussis) vaccine. These are sometimes called cocktail vaccines.
- Multivalent vaccines – Single platform vaccines with multiple antigens designed to generate responses against antigens from distinct pathogens. This term is also used to describe single pathogen vaccines that use multiple antigens from the same pathogen.

Vaccinating against multiple pathogens by combining individually successful vaccines typically results in robust responses to each vaccine. However, there are instances in which a given vaccine has lower efficacy when delivered in a cocktail or multi-pathogen formulation compared to when delivered separately, a phenomenon known as “vaccine interference” (12).

Vaccine interference is a broad umbrella term, as lower efficacy of a vaccine when combined can result from multiple mechanisms of interference. The mechanisms of interference may not be apparent without testing specifically aimed at understanding the mechanism, increasing the numbers of controls and therefore the cost of experiments. Since testing vaccines in animal models prioritizes moving forward efficacious vaccine strategies over understanding poorly performing combinations, understanding why certain antigens can be combined successfully while others interfere requires extrapolation from known mechanisms, as well as understanding antigens, platform technologies and the animal models used in the vaccinations. This review will establish the known mechanisms of vaccine interference, discuss the existing literature on multi-pathogen vaccines in biodefense in the context of their different vaccine platforms and experimental conditions, and provide recommendations for predicting and testing interference in multi-pathogen vaccines. To highlight the phenomena, we will examine multi-pathogen vaccines that include the Marburg virus glycoprotein antigen, and discuss potential mechanisms of interference affecting this antigen.

## 4 Mechanisms of vaccine interference

While there are well established examples of vaccines losing efficacy when combined, the underlying causes of the interference can be difficult to determine. There are multiple proposed mechanisms for how both combined and multivalent vaccines can interfere with one another. The proposed mechanisms can be grouped into three categories; epitope competition, immune response polarization and resource competition. These mechanisms are specific to the vaccine platform, as shown in **Table 1** (13, 14). However, the study of vaccine interference is limited and the level of experimental evidence supporting each of these mechanisms varies considerably.

**Table 1:** Mechanisms of vaccine interference by vaccine platform.

Platform	Vulnerability to vaccine interference:							Logistical features:	
	Transcriptional competition	Ribosome competition	Epitope competition CD8+	Epitope competition CD4+	B-cell epitope competition (antibodies)	Immune polarization	Cell entry/uptake	Scalability	Thermostability
Adenovirus vector	High — uses host transcriptional machinery	High — uses host translation and ribosomes	High— Infected cell MHC class I display. Multiple antigens from same vector highest	Moderate— Cross-presentation or antigen uptake from infected cells, no antigen epitopes incorporated into virion	Moderate— antigens on infected cells/cell debris bind B cells	High — vector causes Th1 polarization alone	High — requires cell uptake for production of antigen	Moderate–high — scalable cell-culture production	Low–moderate — often require freezing
Rhabdovirus vector	None—No transcription, RNA- based	High — uses host translation and ribosomes	High— Infected cell MHC class I display. Multiple antigens from same vector highest	Moderate-high— antigen epitopes are incorporated into virion, can be presented by antigen presenting cells	High — antigen requires no infection to interact with B cells	High — vector causes Th1 polarization alone	High — requires cell uptake for production of antigen (may produce weak B cell response without)	Moderate–high — scalable cell-culture production	Low–moderate — often require freezing
Vaccinia vector	High — uses host transcriptional machinery	High — uses host translation and ribosomes	High— Infected cell MHC class I display. Multiple antigens from same vector highest	Moderate-high— antigen epitopes are incorporated into virion, can be presented by antigen presenting cells	High — vector has antigen on virion, interacts with B cells	High — vector causes Th1 polarization alone	High — requires cell uptake for production of antigen (may produce weak B cell response without)	Moderate–high — scalable cell-culture production	Low–moderate — often require freezing
Bacterial (intracellular) vector	None—Uses bacterial transcriptional machinery	None—Uses bacterial translational machinery	High— Displayed on infected cell MHC class I. Highest when multiple antigens from same vector	Moderate-high— Cross-presentation or antigen uptake from infected cells, antigen epitopes can be incorporated into bacterium	Moderate- high — vector can have antigen on bacterium, interacts with B cells	High — vector causes Th1/Th17 polarization alone	High — requires cell uptake for production of antigen (may produce weak B cell response without)	Moderate–high — scalable cell-culture production	Low–moderate — vectors can be relatively fragile
DNA	High — uses host transcriptional machinery	High — uses host translation and ribosomes	High— Displayed on infected cell MHC class I	Moderate— Cross-presentation or antigen uptake from infected cells, no antigen epitopes in plasmid	Moderate— antigens on infected cells/cell debris bind B cells	Moderate — can cause Th1 polarization through nucleic acid sensing	Moderate— requires cell uptake for production of antigen, but uptake is non-specific	High — fermentation manufacture and purification can occur at scale	Moderate — more stable than mRNA
mRNA	None—No transcription, RNA- based	High — uses host translation and ribosomes	High— Displayed antigens on MHC class I	Moderate— Cross-presentation or antigen uptake from infected cells, no antigen epitopes in mRNA	Moderate— antigens on infected cells/cell debris bind B cells	Moderate — can cause Th1 polarization through nucleic acid sensing, but RNA modifications may reduce immunogenicity	Moderate— requires cell uptake for production of antigen, but uptake is non-specific	High — fermentation manufacture and purification can occur at scale	Low–moderate — Not stable, requires freezing
Protein-based	None—No transcription/replication	None—No transcription/replication	Moderate— Requires cross-presentation to MHC class I	Moderate-high— uptake and presentation by antigen presenting cells likely	High — antigen requires no infection to interact with B cells	None—antigen and adjuvant driven polarization not vector specific	Moderate— weak B cell only response without uptake, but uptake non-specific by APCs	High — recombinant expression systems can support large-scale, standardized manufacturing	Moderate–high — purified proteins can sometimes be formulated for improved stability
Glycoconjugate	None—No transcription/replication	None—No transcription/replication	None — if carrier protein is the same	None — if carrier protein is the same	High — antigen requires no infection to interact with B cells	None—antigen and adjuvant driven polarization not vector specific	Moderate— weak B cell only response without uptake, but uptake non-specific by APCs	Moderate—conjugation and purification can add complexity	Moderate–high —can be stored in refrigerated formulations
Live attenuated virus	Moderate —Specific to virus (only DNA-based viruses affected)	Moderate — may uses host translation depending on attenuation	Moderate- high— Displayed on infected cell MHC class I if infectious	Moderate-high— uptake and presentation by antigen presenting cells likely	High — antigen requires no infection to interact with B cells	Pathogen-specific polarization	High — requires cell uptake for production of antigen (may produce weak B cell response without)	Moderate — biological propagation can be scaled	Moderate — requires cold-chain storage
Inactivated	None—No transcription/replication	None—No transcription/replication	Moderate— Requires cross-presentation to MHC class I	Moderate-high— uptake and presentation by antigen presenting cells likely	High — antigen requires no infection to interact with B cells	Pathogen-specific polarization	Moderate— weak B cell only response without uptake, but uptake non-specific by APCs	Moderate — biological propagation can be scaled	Low–moderate — requires cold-chain storage

Platforms that deliver the antigen as a protein, or result in extracellular/soluble protein release, are processed through the exogenous pathway and presented on MHC class II of antigen-presenting cells. MHC class II epitopes activate CD4+ T cell help, which can elicit antibody maturation. Platforms that deliver antigens for expression inside the cell, such as nucleic acid platforms and viral vectors, will be processed through MHC class I presentation and result in CD8+ T cell activation. Cross-presentation bridges these two pathways, but eliciting robust cross-presentation to generate CD8+ T cells is a challenge across vaccine development (15, 16). Understanding where the antigens interact with the immune system is essential for understanding which mechanisms of vaccine interference are likely to be most relevant. While the mechanisms are discussed as discrete phenomena, each platform has antigens that interact with the host at multiple points, and so interference can have synergistic and overlapping mechanisms.

### 4.1 Epitope competition

In epitope competition, peptide epitopes that are most efficiently displayed on major histocompatibility complex (MHC) molecules are preferentially recognized by the T cell receptors (TCRs) on T cells and dominate the response, generating the largest proportion of specific T cells (13). This immunodominance is accomplished by preferential binding of certain peptides to specific MHCs. Epitope competition has been most studied in cytotoxic T cell (CD8+) responses with MHC class I presentation of peptides, but has also been observed in helper T cell (CD4+) responses with epitopes presented on MHC class II molecules (17). In both cases, the protein must be processed for display on the MHC, but there is conflicting evidence about the importance of the “context” of the peptide in determining immunodominance of specific peptides. The peptide context refers to the tertiary structure, protease susceptibility, and solvent accessibility of the peptide within the larger protein (18). Specific proteolysis of regions surrounding immunodominant peptides has been shown to potentiate antigen presentation (19), but other studies have suggested CD4+ immunodominant peptides in a protein remain the same, independent of their context within the protein (18).

In influenza vaccination, epitope competition may result in a limited response to the conserved stem of hemagglutinin compared to the rapidly mutating head region because of immunodominant peptide presentation (20, 21). In multi-pathogen vaccines, the highest affinity or most readily presented epitopes are the ones that will be presented to T cells (**Figure 1**). Such presentation can impair the responses to an otherwise immunogenic antigen when it is combined with a vaccine that contains an epitope more readily presented on specific MHCs.

**Figure 1:**
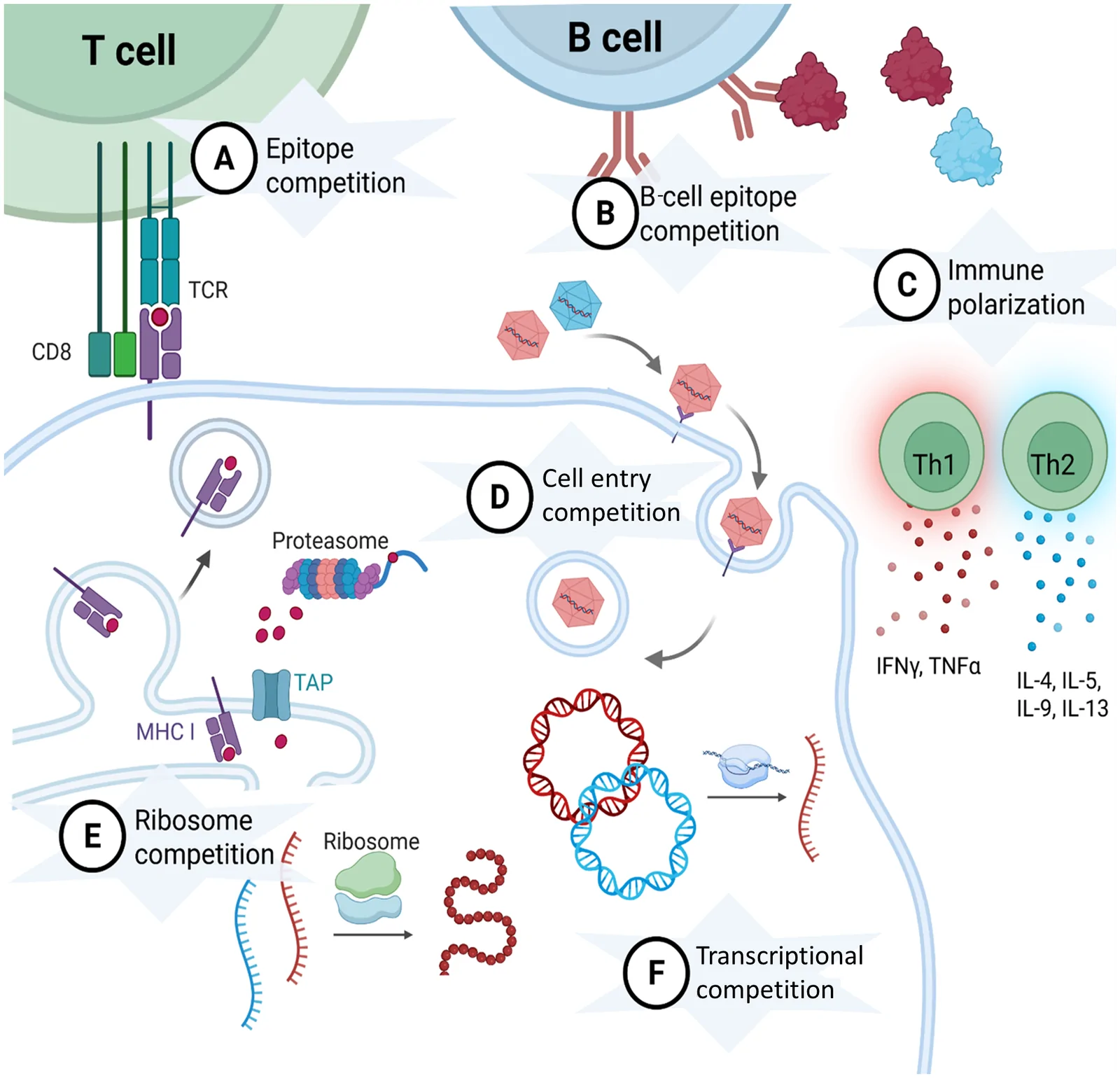
Schematic of proposed mechanisms of vaccine interference when two individually efficacious vaccines are combined. **(A)** Epitope competition through proteolytic processing, transport and MHC binding, and T-cell receptor recognition. **(B)** B-cell epitope competition where B-cell receptors bind more strongly to certain protein motifs and expand preferentially. **(C)** Immune polarization where one vaccine impairs the other. **(D)** Cell entry/uptake competition causes the preferential expansion of some variants within the host at the expense of others. **(E)** Ribosome competition in translation. **(F)** Plasmid competition, focused on transcriptional competition for resources.

Platforms that result in antigens being processed for MHC presentation in the same cell are more likely to be impacted by T cell epitope competition. Vaccine platforms that express multiple vaccine antigens from a single template, such as multivalent viral vector, DNA or mRNA vaccines, will have multiple antigens in the same cell loading epitopes onto MHC class I, making them vulnerable to interference through MHC competition. Additionally, vaccine platforms with fusion proteins as antigens, such as multivalent subunit proteins (or a platform encoding fusion proteins) are vulnerable to MHC class II competition in antigen presenting cells. In both cases, the presence of multiple antigens in the same cell allows for the competition to occur on the MHC-presentation level. However, the delivery of multiple vectors encoding separate antigens may be least vulnerable to epitope competition as the separate vectors may not enter the same individual cell (and for some vectors, uptake of one vector decreases the likelihood of uptake of another (22)). Therefore, separate plasmids, adenovirus or rhabdovirus vectors are less likely to experience MHC-mediated epitope competition.

Immunodominance has also been documented in antibody responses, where some regions of antigenic proteins are better able to bind a wider array of randomly generated antibodies, so B cell populations against them are preferentially expanded (23). For multi-pathogen vaccines, B cell epitope preference is hypothesized to lead to impaired responses to one or more pathogens in the vaccine (**Figure 1B**). Unlike the MHC-binding epitopes, where the competition can only occur if multiple epitopes exist in the same intracellular compartment, the antibody binding occurs outside the cell meaning that each vaccine platform can be impacted by antibody epitope competition. It is not clear how much the spatial proximity of the multiple epitopes competing for B cell binding impacts the competition (23, 24).

### 4.2 Immune response polarization

Different effector mechanisms are triggered during an immune response to combat a specific pathogen or threat, and the selection of one of these is known as immune polarization. Classically, T helper 1 (Th1) responses target intracellular pathogens, while T helper 2 (Th2) responses are necessary to control extracellular pathogens and promote tissue repair (25). The skewing of the immune response by one vaccine or adjuvant may be undesirable for another component in combination vaccines (**Figure 1C**).

In the childhood DTaP vaccine, the diphtheria, tetanus, and acellular pertussis vaccines are combined successfully. However, addition of the *Haemophilus influenzae* type b polysaccharide vaccine (Hib) to the mix resulted in a reduced Hib response when compared to vaccines administered separately (26), as the vaccine interference between DTaP and Hib resulted in a 10-fold drop in mean concentration of Hib antibodies (27). It has been speculated that the reduction in Hib responses when combined with DTaP is a result of a skewing towards a Th2 response from the aluminum-based adjuvant in the combination (28). In contrast, the whole cell diphtheria-tetanus-pertussis whole-cell pertussis (DTwP) vaccine delivered with Hib did not show a reduction in antibody titers against Hib (29) and when delivered alone, Hib vaccination results in primarily a Th1 response. Additionally, in certain cases, the specific chemical or physical interactions between components of the vaccines might alter the conformation or presentation of one of the vaccines, such as the physical incompatibility of one vaccine with the adjuvant of another, such as delicate conjugate antigens interacting with aluminum adjuvants (30). The impact of changes in immune responses to an antigen as a result of combining vaccines or multiple antigens is not necessarily negative. In malaria vaccines, the addition of a more immunogenic plasmid potentiated the antibody response to a plasmid with a poorly immunogenic antigen, indicating an adjuvant-like effect of the additional antigen (31).

While the antigens of a vaccine influence the polarization of the immune response, vaccine platforms also generate characteristic immune polarization. In general, protein-based vaccines such as virus-like particles and subunit vaccines, require specific adjuvants to elicit CD8+ T cell responses, as cross-presentation is required for the antigen to be displayed on MHC class I, and tend to have an antibody-dominated response (32). Viral vector vaccines induce the most robust CD8+ T cell responses, as the immune response to the vector itself is conventionally CD8+ T cell mediated. However, there are differences between different viral vectors, with rhabdovirus vesicular stomatitis virus (VSV) vector Ebola virus (EBOV) vaccines, which both encodes for and carries the EBOV glycoprotein, eliciting less robust CD8+ responses than adenoviral vectors, which just encode the EBOV glycoprotein (10, 33). The success of mRNA vaccines for SARS-CoV-2 has led to examination of how mRNA packaged in lipid nanoparticles (LNPs) elicits strong CD8+ T cell responses, and has demonstrated multiple cross-presentation pathways are engaged upon vaccination to allow for CD8+ T cell activation (34). However, examples of immune polarization interference are most likely to occur in combination vaccines where different platforms are combined, as with DTwP and Hib (35), or where the antigens are driving a specific skewing of the immune response as with malaria plasmid vaccines (31).

### 4.3 Resource competition

Competition for resources between different vaccines should also be considered in combination vaccines (**Figures 1D–F**). In transfections *in vitro*, plasmid competition is well documented, where two co-delivered plasmids compete for resources in the cell to allow for protein expression (36) (**Figure 1D**). The phenomenon is not limited to plasmids with different backbones; a P_CMV_-EGFP plasmid and a P_CMV_-luciferase plasmid have been shown to directly compete in a co-transfection where increasing P_CMV_-EGFP plasmid resulted in a decrease in luminescence from luciferase (37). In a separate study, competition for resources between two different plasmid-expressed proteins was found to occur at the transcriptional level with increasing levels of one plasmid leading to reduced expression from a competing plasmid (38). It is not clear whether competition for resources is unique to the tightly controlled *in vitro* conditions or if it is also the cause of vaccine interference seen *in vivo* in multi-pathogen DNA vaccines discussed below.

Resource competition in translation, or ribosome competition, has been studied less than transcriptional competition. Ribosome competition and the preferential translation of specific mRNAs over others is well established with endogenous mRNAs within the cell. However, the extent to which this preference could lead to a reduction in translation levels for the worse performing mRNA in a vaccine is not clear, although mathematical models to predict the phenomenon exist (39, 40). Additionally, since mRNA vaccines are an emerging technology, research on competition between mRNA vaccines is limited (**Figure 1E**). New technologies to study the factors influencing preferential translation are constantly evolving, particularly within cell-free systems (41, 42), and may allow for identifying the point at which competition occurs in the multi-stage process.

Competition between replicative viruses can cause the preferential expansion of some variants within the host at the expense of others (**Figure 1F**), which may impact viral vector vaccines. Viral competition occurs either when there are differences in uptake and replication efficiency between the two viruses (for replication-competent vaccines), or if one of the two variants is less targeted by the immune system allowing that variant to express more antigen (43).

Platforms that rely on nucleic acids to encode multiple antigens of interest, including viral vectors, DNA and mRNA vaccines, could all be impacted by resource competition. DNA-based vectors (e.g. adenovirus and vaccinia) and plasmids may be impacted by both transcriptional and ribosomal competition, compared to only ribosomal competition for mRNA vaccines and RNA-based vectors (e.g. VSV vectors).

### 4.4 Risks of sub-optimal vaccination

While vaccines are a staggeringly successful public health story, with immunization programs worldwide saving an estimated 154 million lives since 1974 (44), there are risks associated with poor vaccines. In addition to a lack of protection resulting in disease, there are other consequences that can occur if vaccinations are sub-optimal, which is an important consideration in the context of poor immunogenicity in multi-pathogen vaccines. For example, in dengue virus, antibody-dependent enhancement of disease occurs when non-neutralizing antibodies elicited by the vaccine increase disease severity (45). There is also the possibility that vaccination can cause more severe disease than not vaccinating, as is the case with the accelerated filovirus disease progression and “early-death” phenomenon, where non-protective filovirus vaccinations may result in earlier death than in unvaccinated controls, although the mechanism for the phenomenon is unclear (32, 46, 47). The potential for generating sub-optimal responses in multi-pathogen and combination vaccines underscores the importance of using assessment methods that allow direct comparison with their monovalent counterparts during development.

### 4.5 Pre-clinical experimental data and statistical significance

Due to ethical and financial considerations around animal use, and particularly nonhuman primate (NHP) use, there is a limit to the number of animals included in vaccine studies (48). An experiment using a small number of animals may not detect a statistically significant difference (49). It may be sufficient in some experiments to demonstrate efficacy of a multi-pathogen vaccine compared to a placebo/untreated control in a lethal disease model. For the purposes of this review, the most relevant comparison is the difference between a monovalent, single antigen vaccine and the multi-pathogen vaccine counterpart, but comparisons between two efficacious treatments require more animals to show significant differences due to the reduced effect size (49). Therefore, we will discuss some trends in vaccine data that are not statistically significant.

## 5 Vaccine platforms

### 5.1 Adenovirus viral vectors

Replication-incompetent adenovirus (Ad) vectors are a vaccine platform that allows for the delivery of genes encoding antigens. Licensed Ad vector vaccines include adenovirus 26 (Ad26) expressing the glycoprotein (GP) of EBOV (33) and SARS-CoV-2 (50). As a DNA-virus vector, Ad vectors can be impacted by vector competition (if antigens are expressed by multiple separate Ad vectors); transcriptional and ribosomal competition as the antigens are transcribed and translated in the cell; and CD8+ T cell epitope competition when antigens are presented in the cell. Ad vectors expressing soluble or extracellular antigens may also be impacted by antibody epitope competition. Immune response polarization challenges are likely to be mediated by the antigens, as any polarization incompatible with a specific vaccine that was caused by the Ad vector would be evident in a monovalent Ad formulation. Multi-pathogen Ad vector vaccines in development are summarized in **Table 2**.

**Table 2:** Adenovirus vector vaccines.

Pathogens	Vaccine name & platform	Antigens expressed on same vector?	Model	Comparison to monovalent/single formulation?	T cell responses	Antibody responses	Survival in challenge?	Evidence of vaccine interference?
Adenovirus vector vaccines								
EBOV, MARV and SUDV (51, 52)	Ad26/Ad35 adenovirus vectors with EBOV, SUDV and MARV GP (Ad26.Filo)	Each antigen on own vector, co-delivered	*Cynomolgous Macaques*	Yes, compared to monovalent	Not tested	*IgG responses:*	EBOV-Ad26/35 = trivalent (low dose)	Yes, some differences in antibodies elicited in the monovalent EBOV compared to trivalent and higher EBOV survival
						EBOV-Ad26/35 > trivalent/tetravalent	EBOV-Ad26/35 < trivalent (higher dose)	
						SUDV-Ad5 < trivalent	SUDV-Ad5 < trivalent	
						MARV-Ad26/35 > tetravalent (high dose)	MARV-Ad26/35 = tetravalent (high dose)	
	Ad26.Filo with MVA.Filo boost	Each antigen on own vector, co-delivered	Human trial	Yes, compared to Ad26.EBOV (monovalent)	Not tested	Comparable anti-EBOV antibodies and neutralizing antibodies to monovalent	Human trial	No
EBOV and SUDV, MARV and LASV (53)	ChAdOx1 viral vector (boost with MVA-tetra (filo/Lassa)	Bivalent vectors, EBOV/SUDV (biEBOV) and LASV/MARV (biLAMA) co-delivered	CD-1 mice	Yes, compared to 4 monovalent constructs, coadministered	*IFNy responses:*	*IgG responses:*	Not tested	Yes, antibodies elicited in the biEBOV and biLAMA higher than in SUDV monovalent and LASV monovalent respectively
					biEBOV = EBOV-ChAdOx1	biEBOV = EBOV-ChAdOx1		
					biEBOV > SUDV-ChAdOx1	biEBOV = SUDV-ChAdOx1		
					biLAMA > LASV-ChAdOx1	biLAMA = LASV-ChAdOx1		
					biLAMA = MARV-ChAdOx1	biLAMA = MARV-ChAdOx1		
			BALB/c mice	Yes, compared to 4 monovalent constructs, coadministered	*IFNy responses:*	*IgG responses:*	Not tested	Yes, antibodies elicited in the EBOV higher than biEBOV
					biEBOV < EBOV	biEBOV = EBOV-ChAdOx1		
					biEBOV = SUDV	biEBOV = SUDV-ChAdOx1		
					biLAMA = LASV	biLAMA = LASV-ChAdOx1		
					biLAMA = MARV	biLAMA = MARV-ChAdOx1		
			IFNAR-/- mice	Yes, compared to 4 monovalent constructs, coadministered	Not tested	*IgG responses:*	*Survival (IFNAR-/- mice):*	No
						biEBOV = EBOV-ChAdOx1	biEBOV = EBOV-ChAdOx1	
						biEBOV = SUDV-ChAdOx1	biEBOV = SUDV-ChAdOx1	
						biLAMA = MARV-ChAdOx1	biLAMA = MARV-ChAdOx1 (n.s. due to low lethality challenge)	
						Not measured for LASV	biLAMA = LASV-ChAdOx1 (viremia)	
			Hartley Guinea Pigs	Yes, compared to co-delivered EBOV-ChAdOx1 and SUDV-ChAdOx1	Not tested	Not tested	100% survival in EBOV challenge from biEBOV and monovalent EBOV-ChAdOx1	No
EBOV and SUDV (54)	ChAdOx1 viral vector	Bivalent vector EBOV/SUDV (biEBOV)	*Cynomolgus macaques*	No monovalent controls	No or minimal SUDV specific T cell responses	Robust IgG responses and neutralization of SUDV-VSV	0% survival in SUDV challenge	No monovalent controls
EBOV and SUDV (55)	ChAdVax (Ad5 based)	Bivalent vector EBO7	*Cynomolgus macaques*	No monovalent controls	Not tested	Robust total antibody responses to SUDV and EBOV	100% survival in SUDV challenge, 100% survival in EBOV challenge	No monovalent controls

A regimen with a heterologous Ad26-Marburg virus GP (MARV GP) prime boosted with a modified vaccinia Ankara (MVA-filo) vector encoding transgenes for MARV GP, EBOV GP, SUDV GP, and/or Taï Forest (TAFV) GP mixed in a 1:1:1:1 ratio had 100% efficacy against lethal MARV challenge in NHPs. Complete protection was maintained in the multi-pathogen and monovalent MARV-only vaccine groups (51). The mean serum titer of anti-MARV GP antibody and IFN-**γ** responses were highest in the animals receiving the monovalent (Ad26-MARV) vaccine, but this was not statistically significant and only two animals were in the monovalent-MARV group. Protection was 50% for EBOV challenge after the same Ad26 multi-pathogen vaccine or in the monovalent Ad26-EBOV-only vaccinated group (2/4). Clinical signs, antibody titers, and IFN-γ responses were comparable between the two groups, suggesting no interference with the EBOV responses caused by Ad-SUDV or Ad-MARV in the combined vaccine (51).

A first-in-human (Phase 1) safety study using the trivalent Ad26.Filo vaccine (MARV GP, EBOV GP, SUDV GP) discussed above, followed by the approved MVA-filo as the boost showed good safety profiles (52). While monovalent SUDV and MARV vaccines were not tested for comparison, monovalent Ad26-EBOV was compared to the trivalent formulation in a regimen where the monovalent or trivalent was given as the prime and followed with an MVA-filo boost. The anti-EBOV GP antibodies and neutralizing antibodies were comparable between the monovalent Ad26-EBOV and the trivalent Ad26.Filo.

The ChAdOx1 modified chimpanzee adenovirus vector, used in a SARS-CoV-2 vaccine licensed during the COVID-19 pandemic, has also been tested as a multivalent multi-pathogen filovirus vaccine carrying genes for SUDV, MARV, and EBOV GPs [ChAdOx1-triFilo (56)]. The multivalent ChAdOx1-triFilo vaccine was initially optimized for directionality and insertion loci expression cassettes, which influenced expression levels of the GPs. A specific construct was selected, triFilo(2A), which had good expression of EBOV and SUDV GPs and moderate expression of MARV GP (compared to the respective monovalent filovirus GP ChAdOx1 construct expression). The triFilo(2A) vaccine was fully protective in a guinea pig EBOV challenge, inducing cellular and humoral immunity against both SUDV and MARV (56). The authors compared the ChAdOx1-triFilo multivalent vaccine to a combination of the three monovalent vectors (ChAdOx1-EBOV, ChAdOx1-SUDV, ChAdOx1-MARV) delivered together. There were significant reductions in EBOV, SUDV, and MARV humoral and cellular immunity with some of the alternate expression cassettes for the triFilo construct compared to the monovalent single GP combination, but the results mirrored the western blot expression of GPs from those vectors, suggesting that differential antigen production caused the differences in immunogenicity (56).

A modified version of the vaccine where two bivalent ChAdOx1 constructs encoding EBOV/SUDV GPs (ChAdOx1-biEBOV) and LASV G/MARV GP (ChAdOx1-biLAMA) has been developed (53). When compared to a mix of all four monovalent vaccines in a single shot, lower T cell responses were observed against the EBOV antigen when using the two bivalent vaccines together. To address this, the two bivalent ChAdOx1-biEBOV and ChAdOx1-biLAMA formulations were administered at different anatomic sites with no evidence of immune competition between vaccine-encoded antigens (53). ChAdOx1-biEBOV is now moving into first-in-human testing, although it did not protect NHPs in a recent SUDV challenge after a prime-boost regimen (57–59). Interestingly, the triFilo(2A) vaccine and the monovalent ChAdOx1-SUDV GP also failed to elicit neutralizing antibodies against SUDV in CD-1 mice (56). The examination of GP-specific responses in Ad vaccines may be a useful metric in predicting vaccine efficacy, as a logistic model has suggested a strong correlation between anti-GP humoral responses to Ad vaccines and survival in filovirus challenge (52, 60).

### 5.2 *Rhabdoviridae* viral vectors

*Rhabdoviridae* viral vectors use viruses in the *Rhabdoviridae* family (bullet-shaped, negative-sense single-stranded RNA (ssRNA) viruses) to express vaccine antigens. *Rhabdoviridae* vectors, such as vesicular stomatitis virus (VSV) and rabies virus (RABV), may both express the transgene and carry the vaccine antigen on the viral envelope, unlike the Ad vectors which carry only the genetic sequence in the virions themselves. Multi-pathogen rhabdovirus vaccines may be impacted by viral vector competition if combining two vectors; ribosomal competition as the antigens are translated in the cell; and CD8+ T cell epitope competition when antigens are presented in the cell. More so than Ad vectors, rhabdovirus vectors are likely to be affected by antibody epitope competition as many copies of the antigen are present on the virion surface of the vector, and they exist as arrays on the virion giving high avidity to B-cells that bind them.

*Rhabdoviridae* vector-based vaccines are summarized in **Table 3**. Some constructs delete the native VSV or RABV glycoprotein (G) to express the membrane protein of an enveloped virus in its place, allowing the construct to mirror the target virus in tropism and binding (66).

**Table 3:** Rhabdovirus vector vaccines.

Pathogens	Vaccine name & platform	Antigens expressed on same vector?	Model	Comparison to monovalent/single formulation?	T cell responses	Antibody responses	Survival in challenge?	Evidence of vaccine interference?
Rhabdoviridae viral vectors								
MARV, EBOV, SUDV, and BDBV (61)	rVSV-filo Quadrivalent panfilovirus VesiculoVax vaccine	rVSV-MARV, EBOV, SUDV and BDBV separate vectors, coformulated	*Cynomolgus macaques*	No monovalent controls	Upregulation of CD8+ T cells by transcriptional analysis	Early (IgM) and late (IgG) responses to each of the viruses in the multivalent formulation	100% survival BDBV, SUDV and MARV and 80% survival in EBOV challenge	No monovalent controls
MARV, EBOV and SUDV (62)	*rVSV-filo*	Single rVSV vector	BALB/c mice	Monovalent controls with all antigens	*ELISpot IFN-γ:* Monovalent controls> rVSV-filo	*IgG titers:* rVSV-MARV> rVSV-filo (end)	No challenge	Yes, monovalent MARV and EBOV both higher antibodies and IFN-y compared to trivalent
						rVSV-EBOV> rVSV-filo (early)		
						rVSV-SUDVV= rVSV-filo		
			*Cynomolgus macaques*	No monovalent control	ELISpot IFNy induction against all 3 filoviruses in 6/15 macaques	Antibodies elicited against each GP	100% survival in each EBOV, MARV and SUDV challenge	No monovalent controls
EBOV, SUDV, MARV and LASV (63)	*rVSV-filo (Vesiculovax)*	EBOV/SUDV/MARV on same vector, LASV on separate vector and combined	*Cynomolgus macaques*	No monovalent control	*ELISpot IFN-y*: LASV GP–specific (~89%); EBOV/ SUDV GP–specific (~83%); MARV GP–specific (50%)	Neutralizing antibodies against all antigens	100% survival in all challenges	No monovalent controls
EBOV, SUDV, MARV and LASV (64)	Attenuated rabies vaccine strain SAD B19 vector	trivalent (FILORAB1-3) single vector and combined (FILORAB1–3 and LASSARAB) (tetravalent)	*Cynomolgus macaques*	Compared to single antigens on same vector	Not tested	EBOV, SUDV, LASV and MARVendpoint titers: Trivalent and tetravalent = monovalent	No challenge	No, antibodies elicited comparable in monovalent and multivalent
CHIKV and ZIKV (65)	rVSV vector	Single rVSV vector	A129 mice	Compared to monovalent rVSV-CHIKV	Not tested	*Neutralizing antibodies*: rVSV-CHIKV/SIKV = rVSV-CHIKV	100% survival in CHIKV challenge; 100% protection from viremia ZIKV challenge	No, survival comparable in monovalent and multivalent

The recombinant VSV (rVSV) monovalent vaccine for EBOV, Ervebo, is a licensed vaccine, and several *Rhabdoviridae* viral vector vaccines are in pre-clinical development as multi-pathogen vaccines. The quadrivalent VesiculoVax vaccine is made of four live, attenuated, replication-competent rVSV vectors that express the EBOV, SUDV, MARV, and LASV GPs (63). There was no direct comparison to monovalent formulations; however, 100% protection was achieved for SUDV, EBOV, MARV and LASV challenge after two doses. LASV vaccination introduces an additional challenge due to the presence of different lineages of LASV, meaning vaccination with one lineage may not result in cross-protection against another. The quadrivalent VesiculoVax tested heterologous protection (lineage IV vaccination, lineage II challenge) and demonstrated 100% survival after challenge with the heterologous strain (63). The VesiculoVax LASV construct has since been used individually to generate broadly protective LASV responses after only a single dose 3 to 7 days post-vaccination, but no comparison is available with the rapidity of protection from the quadrivalent formulation (67).

Earlier work from the same group compared monovalent and trivalent (VesiculoVax without LASV GP) formulations and showed that while the SUDV and EBOV GP-specific IgG responses were similar from the monovalent and trivalent formulations after a single vaccine dose in BALB/c mice, the MARV GP-specific immune responses were significantly lower after the trivalent formulation vaccine. The reduction in responses was seen in both IFN-γ ELISpot assay testing for specific T-cell responses and anti-MARV GP antibody titers (62). The T-cell and antibody responses did not suggest changes in immune polarization away from Th1 responses towards Th2 responses against MARV in the trivalent, but instead reflected a decrease in the magnitude of the responses.

Inactivated RABV-vectored multivalent vaccine cocktails against EBOV, SUDV, MARV (FILORAB1–3), and LASV (LASSARAB1) GPs induced stable filovirus and LASV antigen-specific immunity for up to 1 year in cynomolgus macaques, with no significant differences between any antibody titers of monovalent and cocktail formulations of the vaccines (64). The multivalent study did not assess metrics of protection outside antibody titer, but a later monovalent LASSARAB-only study showed the single pathogen vaccine resulted in protection in LASV challenge (68).

An rVSV that expresses the CHIKV envelope polyprotein (E3-E2-6K-E1) in place of the VSV glycoprotein (G) and also expresses the membrane-envelope (ME) GPs of Zika virus (ZIKV) in the same construct was protective in A129 mouse challenge models against both CHIKV and ZIKV infection (65). Interestingly, the recombinant rVSV-EBOV-GP vaccine has been used as a vector itself, by including genes for antigens from other pathogens in the construct, which is hypothesized to retain the immune cell tropism conferred by the EBOV-GP while expressing the new antigen (69). The rVSV-EBOV-GP vaccine with ZIKV full-length pre-membrane (prM) and envelope proteins (E) was protective in a lethal ZIKV mouse challenge model, although no EBOV challenge was performed using the new construct, and the efficacy of the rVSV-EBOV-GP-ZIKVprME was not compared to a rVSV-G-ZIKVprME (69).

### 5.3 Vaccinia platform vaccines

The vaccinia-based vector systems are an interesting platform, as the vectors themselves are vaccines for poxviruses, including smallpox and monkeypox, and can be used as vaccines for biodefense as summarized in **Table 4**. As with Ad vectors, vaccinia-systems are DNA-virus vectors, and so can be impacted by transcriptional and ribosomal competition as the antigens are transcribed and translated in the cell; and CD8+ T cell epitope competition when antigens are presented in the cell. As vaccinia is a large and complex DNA virus, antigens tend to be expressed in the same vaccinia construct, which would make vector competition irrelevant. When vaccinia is both a vector and a poxvirus vaccine, considering immune polarization is particularly relevant as the immune response to the other antigen(s) must be compatible with the immune response to poxvirus. A thorough review of the vaccinia-based vector systems discusses the applications and development of the vectors for emerging diseases (57).

**Table 4:** Vaccinia vector vaccines.

Pathogens	Vaccine name & platform	Antigens expressed on same vector?	Model	Comparison to monovalent/single formulation?	T cell responses	Antibody responses	Survival in challenge?	Evidence of vaccine interference?
Vaccinia viral vectors								
ZIKV and CHIKV (70, 71)	SCV-ZIKA/CHIK Sementis Copenhagen Vector (vaccinia)	Yes	WT and IFNAR^−/−^ mice	Yes, to SCV-CHIKV	Not tested	SCV-ZIKV/CHIKV ≥ SCV-CHIKV against CHIKV antigen/neutralization	Reductions in viremia after challenge (non-lethal challenge)	No, bivvalent ≥ monovalent in neutralization for CHIKV
			*Rhesus macaques*	No	Not tested	Neutralizing antibodies against ZIKV and CHIKV	Reductions in viremia after challenge (non-lethal challenge)	No monovalent controls
New world arenaviruses (Machupo (MACV), Junín (JUNV) Sabia virus (SABV), Guanarito virus (GTOV) and Chapare virus (CHAV)) (72)	MOPEVAC_NEW,_ Mopeia virus vector (non-pathogenic arenavirus vector)	Yes	*Cynomolgus macaques*	Yes, to monovalent MOPEVAC_MAC_ (Machupo)	Only investigated against MACV, low in both monovalent and multivalent	*IgG titers:* MOPEVAC_NEW_ =monovalent MOPEVAC_MAC_	100% survival against MACV and GTOV challenge	No, multivalent ≥ monovalent antibodies and full protection in MACV challenge for both
SARS-CoV-2, SARS-CoV, and MERS-CoV) and influenza viruses (H7N9 and H1N1) (73)	Tiantan strain of vaccinia virus (rTTV-RBD-HA2)	Yes	C57BL/6 mice	No comparison to monovalent	Against receptor binding domains (RBDs) of the three coronaviruses and to influenza HAs	Against receptor binding domains (RBDs) of the three coronaviruses and to influenza HAs	100% protection against SARS-CoV-2 virus and the influenza A H1N1 and H3N2 virus challenges	No monovalent controls
Anthrax toxin and smallpox (74)	Live attenuated vaccinia PA antigen from anthrax (KVAC-thPA-C7L)and immunoadjuvants IL-15 and Cholera toxin (KVAC-hIL15-C7L and KVAC-CTA1-C7L)	No, anthrax PA on one vector, immunoadjuvant on another vector	A/J mice	No, comparison with or without immunoadjuvant expression	Not tested	*Anti-PA titers:* PA only < Immunoadjuvant+ PA	*B. anthracis* challenge: PA only: 62.5% survival < Immunoadjuvant+ PA: 100% survival	No monovalent controls
						Significant neutralization against vaccinia	No poxvirus challenge	No monovalent controls

The multivalent MVA-filovirus vaccine Mvabea has been approved for use by the European Medicines Agency. This vaccine contains an MVA vector encoding the EBOV GP, SUDV GP, MARV GP, and TAFV nucleoprotein. It is approved when used as the “boost” after a prime with an Ad26-vectored virus expressing the GP of EBOV (33). However, the licensure for this vaccine regimen is based on EBOV protection data (58). Whether the inclusion of the multi-pathogen boost is sufficient to induce strong anti-SUDV and MARV responses has not yet been determined (60).

A trivalent MVA-vector haemorrhagic fever vaccine used antigens optimized for broad cross-reactivity from each of orthoebolavirus GP, orthomarburgvirus GP and Lassa mammarenavirus NP. The trivalent vaccine, HFVac3.v1, was compared to monovalent vaccines with unmodified wild-type EBOV GP, MARV GP and LASV NP, and demonstrated greater binding to selected antigens (BDBV, SUDV, RAVV, MARV GPs and LASV NP) than the monovalent for all antigens except EBOV GP. The differences in antigen binding between HFVac3.v1 with an optimized orthoebolavirus GP most closely related to SUDV GP, and the monovalent vaccine expressing wild-type EBOV GP may be due simply to the differences in the antigen, or be due to vaccine composition and antigenic interference in the multi-pathogen vaccine (59).

The Sementis Copenhagen Vector (SCV) is a vaccinia platform that has been used for CHIKV and ZIKV, and the level of vaccinia virus (VACV) neutralization after two doses of the vaccine is high in NHPs (70), suggesting cross-purpose use is possible. SCV expresses the ZIKV envelope protein and CHIKV structural protein cassette (E1/E2 and capsid) in a single construct (75). This is distinct from many of the viral vector vaccines as each viral particle in the vaccine is multivalent: both CHIKV and ZIKV are present on each particle. The SCV-ZIKA/CHIK multivalent and monovalent SCV-CHIK both induced significant antibody titers and neutralizing antibody responses to CHIKV in mice, with the multivalent vaccine showing higher, but not statistically significant, responses in both. While there was no comparison between the multivalent and a ZIKV monovalent SCV, the SCV-ZIKA/CHIK was protective against ZIKV infection in an interferon alpha receptor knock-out (IFNAR(-/-)) mouse model of infection (70, 71).

The Tiantan strain of vaccinia virus, TTV-752-1, was used to generate a multivalent vaccine rTTV-RBD-HA2 against different coronaviruses (SARS-CoV-2, SARS-CoV, and MERS-CoV) and influenza viruses (H7N9 and H1N1 influenza virus). While there was no comparison to monovalent vaccines, TTV-RBD-HA2 induced antibody responses and specific T cell responses to the receptor binding domains (RBDs) of the three coronaviruses and to group 1 and 2 HAs in mice. The rTTV-RBD-HA2 vaccination protected mice against SARS-CoV-2 virus, the influenza A H1N1 and H3N2 virus challenges, however, ablation of either CD4+ or CD8+ T cells after vaccination resulted in a loss of protection in influenza challenges (73).

A multi-pathogen vaccine using KVAC103 vaccinia as a vector for expression of *B. anthracis* PA, one of the anthrax toxins, demonstrated protection after challenge with anthrax spores. The KVAC103 vaccines highlight the possibilities for further manipulation of the poxvirus vector due to their large genome size; for example, the increased capacity for transgenes allowed for the addition of immunoadjuvants IL-15 and cholera toxin subunit A (non-antigen genes), which increased the desired immune responses and protection after anthrax spore challenge compared to non-adjuvanted KVAC103 (74).

### 5.4 Bacterial vectors

The large genome size and easy genetic manipulation of bacteria make them promising candidates as multi-pathogen vaccine platforms, as summarized in **Table 5**. The bacterium *F. tularensis* live vaccine strain (LVS) *capB* deletion (Δ*capB*) mutant is an attenuated *F. tularensis* strain that is protective in mouse models of tularemia (78), and has been used as a vector to express *B. anthracis* proteins (76). The bacterium *F. tularensis* live vaccine strain (LVS) is an intracellular bacteria that replicates in the cell cytoplasm, but have independent transcription and translation machinery, reducing potential resource competition in cells. As with vaccinia, bacterial vectors are large and complex, meaning that antigens tend to be expressed in a single bacterial construct, making vector competition irrelevant. Intracellular bacteria may be impacted by CD8+ T cell epitope competition when antigens from the bacteria are trafficked for antigen presentation. As the bacteria discussed are both vectors for additional antigens and vaccines for their non-attenuated pathogen, immune polarization is particularly relevant as the immune response to the other antigen(s) must be compatible with the immune response to the bacterial vector.

**Table 5:** Bacterial vector vaccines.

Pathogens	Vaccine name & platform	Antigens expressed on same vector?	Model	Comparison to monovalent/single formulation?	T cell responses	Antibody responses	Survival in challenge?	Evidence of vaccine interference?
Bacterial vector vaccines								
*Bacillus anthracis* and *Francisella tularensis* (76)	Live attenuated *F. tularensis* (rLVS ΔcapB /Ba)	Yes, anthrax PA expressed in LVS *F. tularensis*	BALB/c mice	Compared to anthrax adsorbed antigen vaccine (AVA)	Specific PA and LVS CD4+ T cells	Pa-, LF- and HI-LVS-specific antibodies	*B. anthracis challenge:* rLVS ΔcapB /Ba 100% protection > AVA anthrax control 20% protection *F. tularensis challenge:* Only vectors without *B. anthracis* antigens rLVS ΔcapB compared in challenge	No, performed better than different platform for *B. anthracis* and no monovalent control for *F. tularensis*
*Yersinia pestis* and *Francisella tularensis* (76)	Live attenuated *F. tularensis (rLVS ΔcapB* /Yp)	Yes, *Y. pestis F1 and LcrV* expressed in LVS F. tularensis	BALB/c mice	Compared to unlicensed EV76 *Y. pestis* vaccine	IFN-γ secretion by lung and spleen cells in response to F1 and LcrV	LcrV and F1 serum antibodies elicited	*Y. pestis challenge*: 40% protection rLVS ΔcapB/Yp < EV76 *Y. pestis* vaccine *F. tularensis challenge*: Only rLVS ΔcapB vectors without Y. pestis antigens compared in challenge	No, performed less well compared to different platform for *Y. pestis*, and no monovalent control for *F. tularensis*
*Burkholderia pseudomallei* and *Francisella tularensis* (77)	Live attenuated *F. tularensis (rLVS ΔcapB/Bp (Hcp6-Hcp1-Hcp2))*	Yes, *Y. pestis* Hcp6-Hcp1-Hcp2 expressed in LVS *F. tularensis*	BALB/c mice	Compared to Bp82 *B. pseudomallei (*live vaccine strain*)* vaccine	Not tested	Hcp6 and Hcp1 IgG titers	*B. psuedomallei* challenge: 45% protection IN rLVS ΔcapB/Bp = 30% ID Bp82 vaccine (LVS ΔcapB vector alone produced some protection)	No, performed equivalently well compared to different platform for *B. pseudomallei*

The LVS Δ*capB* vector expressing LF and PA proteins from *B. anthracis* was compared to the licensed anthrax acellular protein-based vaccine (anthrax vaccine adsorbed (AVA)) in a lethal *B. anthracis* challenge. The LVS Δ*capB B. anthracis* vaccine resulted in higher survival in mice (75%) compared to 40% with a 3-dose homologous AVA vaccination. While the AVA vaccine is composed primarily of PA with a lower level of LF and other proteins, the LVS Δ*capB B. anthracis* vaccines expressed LF and PA as a fusion protein and had the same level of expression of each. Interestingly, the LVS Δ*capB* vaccines had significantly higher LF-specific titers, but lower PA-specific titers than AVA, and an apparent polarization towards a Th1 response dominated by high IgG2 titers. LVS Δ*capB B. anthracis* mice also had elevated levels of lung and spleen CD4+ T cells expressing IFN-γ, TNF-α, IL-2, and IL-17A compared with AVA-immunized mice (76), suggesting that bacterial vector vaccines can successfully elicit specific T cell responses. In addition to LVS Δ*capB*, recombinant *Listeria monocytogenes* was used as a vector to express the above antigens and was successfully used as heterologous boosts in combination with LVS Δ*capB* against *B. anthracis.*

The LVS Δ*capB* vector was also used to express a fusion protein of *Y. pestis* F1 and LcrV antigens (LVS Δ*capB* F1-V), and the vaccine was compared to the unlicensed live attenuated *Y. pestis* EV76 vaccine. Neither homologous (LVS Δ*capB*) nor heterologous (LVS Δ*capB* followed by *L. monocytogenes*) prime-boost regimen resulted in more than 50% survival in *Y. pestis* challenge, compared to 100% survival after EV76 vaccine (76). The majority of the IgG antibodies induced by LVS Δ*capB* were LcrV-specific, compared to EV76, which elicited low LcrV-specific but substantial amounts of IgG to F1. However, later dosing improvements showed that the same LVS Δ*capB* F1-V construct generated IgG titers against the F1 antigen comparable to EV76, and 100% protection in *Y. pestis* challenge after 3 doses (79). The rLVS Δ*capB* has been employed successfully to generate melioidosis vaccines expressing *Burkholderia pseudomallei* type VI secretion system proteins Hcp1, Hcp2, and Hcp6, and membrane protein LolC. The rLVS Δ*capB*/*B. pseudomallei* vaccines protected highly sensitive BALB/c mice against lethal respiratory *B. pseudomallei* challenge. Interestingly, the LVS Δ*capB* vector alone provides some protection against *B. pseudomallei* challenge and induces a significant humoral immune response (anti-HI Bp82 antibody titers) compared to sham-vaccinated mice (77).

### 5.5 Other viral vectors

Mopeia virus (MOPV), a non-pathogenic arenavirus, has been modified to express glycoprotein complex (GPC) antigens from the New World arenaviruses Machupo (MACV), Sabia virus (SABV), Guanarito virus (GTOV), Chapare virus (CHAV), and Junín virus (JUNV). The combination of the arenavirus-MOPV vaccines together is called MOPEVAC_NEW_, for Mopeia virus vaccine expressing the New World arenaviruses. MOPEVAC_NEW_ was able to fully protect NHPs against MACV and GTOV infection. Only the monovalent MOPEVAC expressing MACV GPC (MOPEVAC_MAC_) was available for comparison with the multi-pathogen cocktail, but anti-MACV IgG titers after MOPEVAC_NEW_ and MOPEVAC_MAC_ vaccination were comparable (72).

Sindbis virus (SINV), is an alphavirus which can be used a vaccine platform by replacing the scaffolding protein genes with antigens of interest to create a chimera. Chimeric Sindbis-VEEV/EEEV/WEEV viruses have been developed to express structural proteins (capsid and pE2/6k/E1) from VEEV, EEEV, or WEEV in place of SINV structural proteins, followed by inactivation of the attenuated virus to prevent SINV infection (80). Each individual SINV construct expresses either WEEV, EEEV or VEEV structural proteins, which are then combined and delivered together. The combined Sindbis-VEEV/EEEV/WEEV induced a balanced response against all three chimeric viruses, although mice vaccinated with either monovalent (e.g. SINV-VEEV only) or trivalent formulation were protected from mortality against the respective virus. While more mice showed clinical signs of infection in the combined vaccination groups compared to the monovalent, this did not appear to be statistically significant (80). The SINV backbone has also been used to generate combined SARS-CoV-2 and Dengue (DENV1 and DENV2) multi-pathogen vaccines which are protective in mice (81).

Measles virus (MV) is a safe, immunogenic attenuated vector capable of heterologous antigen delivery, with progress of an MV-based Chikungunya vaccine into clinical trials (82) highlighting the versatility of the viral vector. A multi-pathogen MV-EBOV_GP_+MARV_GP_ vaccine was compared to individual MV-EBOV_GP_ and MV-MARV_GP_ vaccines in mice (83). The monovalent MV-EBOV_GP_, induced significantly higher levels anti-EBOV GP IgG antibodies than MV-EBOV_GP_+MARV_GP_, as well as significantly higher neutralizing antibodies. Vaccination with the MV-EBOV_GP_ provides complete protection against lethal mouse adapted (MA)-EBOV challenge, compared to only 3/5 mice protected after MV-EBOV_GP_+MARV_GP_ vaccination. Although replication of the vaccine constructs were assessed *in vitro*, it is possible that the MV-EBOV_GP_+MARV_GP_ construct has poorer antigen expression in the mouse, and this could account for the differences between the bivalent and monovalent vaccines and should be investigated (83).

MOPV, SINV and MV vectors are all RNA-virus vectors, but there are key differences. Each vector could be impacted by T cell epitope competition from CD8+ T cells, and also ribosomal competition during expression of antigens. Unlike the VSV-vectors, MV vectors do not incorporate expressed foreign glycoproteins on the virion surface, and typically only express MV fusion and hemagluttinin on the surface due to matrix protein interactions (84). SINV vectors can express the envelope and capsid proteins of other alphaviruses to generate chimeras, or have non-related antigens encoded but not present as part of the structure (81), with the former more likely to generate strong antibody responses. The MOPV vectors are used to express 5 pathogenic alphavirus GPCs which are combined into a single formulation, making viral vector competition possible, along with ribosomal competition and epitope competition. Specific responses to the vector are not required in these platforms, meaning that immune polarization interference from combining two or more antigens will depend on the antigens expressed.

### 5.6 Subunit and virus-like particle vaccines

Subunit vaccines contain only the protein antigen from the target pathogen, and are a versatile platform used for viruses, bacteria, and toxin targets. Subunit vaccines are already in use against bacterial toxins, including the licensed vaccine against anthrax, AVA (Biothrax) (85). Virus-like particles (VLPs) generally contain protein antigens on a scaffold, with or without a lipid bilayer component. As protein-based vaccines that do not replicate, this category of vaccine is not impacted by resource competition within the cell for ribosomes or transcriptional resources. Although the proteins are primarily presented to CD4+ T cells through the exogenous pathway, successful vaccines may induce CD8+ T cell responses through cross-presentation, which can be antigen-specific or elicited by adjuvants, making T cell epitope competition a possibility. Strong antibody responses are most associated with subunit and VLP vaccines, and so B cell epitope competition is possible. Co-delivered proteins that are separate and fusion constructs may have different susceptibility to epitope competition. Both subunit and VLP vaccines are summarized in **Table 6**.

**Table 6:** Protein and virus-like particle vaccines.

Pathogens	Vaccine name & platform	Antigens expressed on same vector?	Model	Comparison to monovalent/single formulation?	T cell responses	Antibody responses	Survival in challenge?	Evidence of vaccine interference?
Subunit and virus-like particle vaccines								
EBOV, SUDV and MARV (86)	*Drosophila* S2 produced subunit vaccines and 10 mg CoVaccine HT™	No, individual proteins	*Cynomolgus macaques*	No comparison to monovalent	Not tested	High against SUDV, EBOV and MARV	100% protection in Ebola, and Sudan challenge	No monovalent controls
EBOV and MARV VLP combination (46)	Ebola and Marburg virus- like particles	Yes, virus-like particles produced to carry both EBOV and MARV GP on same VLP	Strain 13 guinea pigs	Yes, to individual VLPs for EBOV and MARV	Not tested	Comparable to EBOV-VLP and MARV-VLP	EBOV challenge: 90% survival combined VLP = 100% EBOV-VLP (n.s.)	No, comparable to monovalent VLP controls for EBOV challenge and antibody titers similar for both MARV and EBOV
*Bacillus anthracis, Staphylococcus aureus, Yersinia pestis*, and *Clostridium botulinum* (87)	F1-V, STEBV. rPA, and BoNT/A(Hc) recombinant protein vaccines	No, individual proteins. Microneedles deliver separately at the skin to prevent interactions	*Rhesus macaques*	No comparison to monovalent proteins	Not tested	Neutralizing antibody titers against anthrax, BoNT/A, and Staphylococcus	*Bacillus anthracis* spores, botulinum neurotoxin A, or staphylococcal enterotoxin B challenge: 100% survival	No monovalent controls
Ricin and *Bacillus anthracis* (88)	Ricin enzymatic subunit (RTA) and dominant negative inhibitor (DNI) from *B.anthracis*	No, individual proteins	Balb/c mice	Yes, to individual RTA and DNI subunits	Not tested	*Antibody titers*: RTA > RTA/DNI DNI > RTA/DNI	5 of 5 survived ricin challenge; 4 out of 5 survived LT challenge	Yes, higher antibody titers in monovalent vaccines than bivalent
*Bacillus anthracis* and *Yersinia pestis* (89)	Single recombinant protein F1 and V from *Y.pestis* and PA from *B. anthracis* (F1mutV+PA)	Yes, fusion protein	BALB/c mice	Yes, compared to F1mutV without PA and PA	Not tested	*F1mutV antibodies*: F1mutV+PA = F1mutV *PA antibodies*: F1mutV+PA < PA	*Sequential Lethal toxin (LeTx) and Yersinia pestis challenge*: 80% protection F1mutV+PA< 90% protection F1mutV *Combined Lethal toxin (LeTx) and Yersinia pestis challenge*: 75% protection F1mutV+PA< 100% protection F1mutV	Yes, poorer performance in challenge with Y.pestis in bivalent than monovalent. Both PA monovalent and bivalent protected in lethal toxin challenge

Multi-pathogen vaccines against seven serotypes of *C. botulinum* neurotoxins (BoNTs), defined by their lack of cross-serotype toxin neutralization, were formulated from the non-toxic carboxyl-terminus of the heavy chain (Hc) domain of the seven BoNTs and delivered together as a vaccine. The BoNT vaccine protected mice from challenge with each of the seven serotypes (90).

A filovirus GP subunit vaccine was delivered as a combination of EBOV, SUDV, and Bundibugyo (BDBV) GPs in a 1:1:1 ratio (Tri-GP) and compared to monovalent vaccines in mice (91). The Tri-GP showed a slight reduction in antibodies to EBOV compared to responses to the individual GPs. There was also a reduction in the BDBV response compared to the monovalent control that reached significance, but only at one time point (day 28 post-vaccination). In addition to the reduction in total antibody titers, there was also a small reduction in SUDV and EBOV specific neutralization in the Tri-GP compared to the EBOV GP and SUDV GP monovalent vaccines. Interestingly, the numbers of IFN-γ spot-forming cells (CD8+ T-cell functional readout) in mice receiving Tri-GP were markedly reduced relative to the responses to the individual GPs combined. Combining Tri-GP with liposomes or lipid nanoparticles recovered the loss of function of the Tri-GP compared to monovalent GPs formulated without liposomes, but the mechanism by which the liposome formulation improving the T-cell outcomes is not known (91).

A monovalent filovirus candidate subunit vaccine containing EBOV GP tested in NHPs demonstrated full protection against a homologous EBOV challenge after three doses and partial protection after two doses (86, 92). Three doses of the subunit vaccine containing SUDV and MARV GP were 100% protective both singularly (25 µg SUDV/MARV GP and 10 mg of the adjuvant CoVaccine HT™) and when delivered alongside the same concentration of EBOV GP (25 µg). There were non-significant reductions in the anti-MARV IgG antibody levels in the combination compared to single pathogen subunit vaccines at early (single dose) time points (86). The same group had previously tested the subunit-adjuvant formulation in both mice (93) and guinea pigs (92), but only the monovalent EBOV GP was 100% protective in a lethal challenge (92).

One consideration for multi-pathogen vaccines is whether long-term storage may alter the immunogenicity of one antigen more than others. A multi-pathogen filovirus (SUDV, EBOV and MARV GP) subunit vaccine was either lyophilized in individual components and combined before delivery, or lyophilized and stored in the single-vial trivalent formulation before efficacy testing in mice (94). The vaccines formulated together and the vaccines formulated separately were stored at 40 °C for 2 years, and resulted in the same immune responses for EBOV and MARV. However, the mix of individually stabilized GPs elicited significantly higher antibody responses than the single-vial trivalent formulation against SUDV GP. While the mechanism by which the different formulations resulted in changes in efficacy is unknown, these data suggest that stability in storage must be assessed in the multivalent formulation as interactions such as aggregations with other antigens may alter longevity (94).

VLP vaccines are non-infectious, non-replicating vaccines that resemble the outer shell of a virus. A combination of EBOV and MARV VLPs was protective against both EBOV and MARV challenge in the guinea pig model. A reduction in EBOV survival in the multivalent compared to the monovalent vaccine was seen, but this was not statistically significant (46). Similarly, Nipah virus G, F and M proteins can be expressed in mammalian cells to generate Nipah VLPs which have been shown to be protective against Nipah virus infection in the hamster model (95). The NiV VLP was used backbone for a multi-pathogen NiV-HeV-EBOV VLP vaccine, expressing NiV M, F, and G, HeV F and G, and EBOV GP (96). The NiV-HeV-EBOV VLP vaccine produced neutralizing antibody titers against pseudovirus EBOV, NiV and HeV, but had poorer responses than a similarly multivalent VSV-pseudovirus expressing the same proteins, particularly to EBOV. The multi-pathogen VLP vaccine was not compared to monovalent VLPs, but incorporation into the VLPs was not uniform across the different antigens, likely due to a combination of expression differences and efficiencies in budding and assembly (96).

Nanoliposomes are a liposome display platform in which His-tagged protein subunits chelate cobalt on cobalt-porphyrin-phospholipid (CoPoP) liposome surfaces, allowing multiple antigenic subunits to be assembled into a single vaccine (97). Recombinant influenza hemagglutinin (HA) ectodomains, the SARS-CoV-2 receptor-binding domain (RBD), and the respiratory syncytial virus (RSV) F ectodomain have been combined using this platform to generate a five-component respiratory virus vaccine that protected against influenza, RSV, and SARS-CoV-2 challenge (97). A CoPoP formulation displaying the SARS-CoV-2 RBD has also demonstrated safety and immunogenicity in a phase 3 clinical trial (98). The platform can incorporate adjuvants to enhance or direct immune responses; including PHAD, a TLR4 agonist, and QS-21, a saponin adjuvant that promotes T-cell polarization and phagocytosis (97). Immune responses to the multivalent formulation were comparable to monovalent vaccines, with similar antigen-specific IgG titers, hemagglutination inhibition (HAI), SARS-CoV-2 pseudovirus neutralization, and RSV neutralization (97).

The inclusion of RSV provides interesting data due to vaccination-related enhanced respiratory disease (ERD). In ERD, RSV vaccination elicits poor neutralizing antibody responses and Th2-skewed immunity promoting immune complex deposition in the lungs and worsening disease outcomes (99). ERD is a relevant consideration for multivalent vaccines, as combining antigens can alter immune polarization, a proposed mechanism underlying interference observed with DTaP and Hib vaccines (35). The authors compared the five-component and monovalent RSV nanoliposome vaccines with formalin-inactivated RSV (FI-RSV), a discontinued vaccine associated with ERD. Unlike FI-RSV, both nanoliposome formulations induced neutralizing antibodies and lacked persistent RSV RNA and peribronchiolar inflammation after challenge (97).

There are a number of protein subunit-based multi-pathogen vaccines for bacteria or toxins in development. *B. anthracis* PA has been combined with antigens from *Y. pesti s (*89), *C. botulinum* (100) and *F. tularensis* (76), in other vaccine platforms. No significant antigenic interference was observed in the *Y. pestis-B. anthracis* subunit vaccine, but there was a slight increase in *Y. pestis* antigen responses when given alone compared to the combined *Y. pestis-B. anthracis* subunit vaccine (89).

A microneedle has been specifically designed to inject four separate recombinant protein vaccines for anthrax (rPA), botulism (BoNT/A(H_c_)), plague (rF1-V), and staphylococcal enterotoxin B (STEBVax). This approach may mitigate some types of vaccine interference that rely on the different vaccines being present in the same cell. In this approach, vaccines are delivered at the same “site” but alongside one another to prevent direct interactions that may be incompatible. The vaccine was 100% effective against otherwise lethal aerosol challenge with *B. anthracis* spores, BoNT A, or staphylococcal enterotoxin B. The final antibody levels for rPA, BoNT/A(H_c_), rF1-V, and STEBVax were similar to previous values for animals receiving individual vaccinations (87).

### 5.7 Nucleic acid vaccines

Nucleic acid vaccines are DNA or RNA based vaccines which encode vaccine antigens and contain no protein components (unlike viral vector vaccines). The majority of the literature on nucleic acid vaccines for biological threat pathogens is on DNA plasmid-based vaccines (5) as summarized in **Table 7**. Nucleic acid vaccines can elicit CD8+ T cell responses by expressing antigens intracellularly, and so expression of multiple antigens can result in resource competition inside the cell, both transcriptional and ribosomal competition. Expression of extracellular or soluble antigens is most likely to elicit antibodies and therefore result in antibody epitope competition, but even intracellular antigens that elicit poor antibodies could be impacted by T cell epitope competition. Host sensing of nucleic acids is able to polarize the immune response towards a Th1 response, but to cause interference two nucleic acid vaccines would have to elicit different immune responses to one another, which would be most likely to occur through the expressed antigen.

**Table 7:** Nucleic acid vaccines.

Pathogens	Vaccine name & platform	Antigens expressed on same vector?	Model	Comparison to monovalent/single formulation?	T cell responses	Antibody responses	Survival in challenge?	Evidence of vaccine interference?
Nucleic acid vaccines								
ZIKV, DENV 1–4, and CHIKV (101)	MMBV( Multi-mosquito borne viruses) DNA vaccine	No, individual plasmids for each antigen combined for delivery	C57BL/6 mice	Yes, compared to each individual plasmid vaccine	*IFNγ ELISpot:* MMBV produced comparable responses to each individual vaccines	100% seroconversion rates against ZIKV, DENV2, and CHIKV but lower against DENV1, 3, and 4	No challenge	No, comparable to monovalent controls
			*Rhesus macaques*	Yes, compared to each individual plasmid vaccine	*IFNγ ELISpot:* MMBV produced comparable responses to each individual vaccines, poorer against CHIKV in both cocktail and individual	80% (CHIKV) to 100% (ZIKV, DENV1-4) seroconversion, equivalent to individual formulations	No challenge	No, comparable to monovalent controls
LASV, EBOV, MARV (101)	Multivalent Hemorrhagic Fever Virus (MHFV)	No, individual plasmids for each antigen combined for delivery	Hartley guinea pigs	Yes, compared to each individual plasmid vaccine	*Not tested*	*MARV IgG:* Individual MARV> Cocktail *EBOV and LASV IgG:* Individual = Cocktail	No challenge	Yes, MARV monovalent has higher antibody responsis than in multivalent. Not explained by expression differences
			*Rhesus macaques*	Not compared to individual plasmids	*IFNγ ELISpot:* MHFV produced antigen specific responses	EBOV and LASV 100% seroconversion, weaker against MARV (80% seroconversion)	No challenge	No monovalent controls
*Bacillus anthracis* and *Clostridium botulinum* (100)	pD4/HCt DNA vaccine (PA-D4(B. anthracis), HCt(C. botulinum))	Yes, single plasmid expressing both antigens	Balb/c mice	Not compared to individual antigens	Not tested	Anti-PA-D4 and HCt antibodies elicited	Not tested	No monovalent controls
			A/J mice	Not compared to individual antigens	Not tested	Anti-PA-D4 and HCt antibodies elicited, Th1/Th2 response to both antigens, but Th2 skewed	50%- 66% protection in challenge compared to 0% in unvaccinated	No monovalent controls
			Guinea Pigs	Not compared to individual antigens	Not tested	Anti-PA-D4 and HCt antibodies elicited, no significant differences at different doses	Not tested	No monovalent controls
*B. anthracis*, EBOV, MARV, and VEEV (102)	DNA expressing PA(B.anthracis, GP (EBOV and MARV) and Env (VEEV)	Separate plasmids combined for delivery	Guinea Pigs	Compared to individual plasmids	Not tested	High antibody titers against each antigen comparable to individual	*EBOV challenge:* EBOV DNA 4/6 > Multi 3/5 *MARV challenge:* MARV DNA 3/6 < Multi 4/6 *VEEV challenge:* VEEV DNA 6/6 > Multi 5/6	Possible; no statistically significant differences but higher survival in EBOV and VEEV compared to multivalent (lower in MARV monovalent)
Puumala (PUUV)and Hantaan (HTNV) (103, 104)	PUUV and HTNV M-segment DNA vaccine (pWRG7077 backbone, non-codon optimized sequences)	Separate plasmids combined for delivery	Hamster	Compared to individual plasmids	Not tested	*Neutralizing antibodies against HTNV:* HTNV individual > Combined *Neutralizing antibodies against PUUV:* PUV individual > Combined	*HTNV non-lethal challenge:* HTNV 88% > Combined 25%	Yes, both PUUV and HTNV monovalent elicited higher neutralizing antibodies
Puumala (PUUV)and Hantaan (HTNV) (105–107)	PUUV and HTNV M-segment DNA vaccine pWRG/HTN-M and pWRG/PUU-M (s2)	Phase 1 clinical trial	Clinical trial	Yes, compared to each individual plasmid vaccine	Not tested	64% HTNV vaccine > 55% combined; 75% PUUV vaccine = 77% combined	Not applicable	Yes, HTNV monovalent elicited higher antibodies than bivalent. PUUV equivalent
	HTNV and PUUV M segment DNA vaccine (gene optimized pWRG/PUU-M (s2) and pWRG/HTN-M(co))	Phase 1 clinical trial	Clinical trial	Yes, compared to each individual plasmid vaccine	Not tested	*Neutralizing antibody responses:* 100% of HTNV or PUUV vaccines > 44% combined vaccine	Not applicable	Yes, both PUUV and HTNV monovalent elicited higher neutralizing antibodies
	PUUV and HTNV M-segment DNA vaccine (gene optimized pWRG/PUU-M (s2) and pWRG/HTN-M(co))	Phase 2a clinical trial	Clinical trial	No	Not tested	80-90% sero-positivity from combined vaccine against HTNV and 73.3-86.7% against PUUV	Not applicable	No monovalent controls

A combination of four DNA vaccines for *B. anthracis*, VEEV, EBOV, and MARV were shown to be equally as effective as the individual vaccines in eliciting specific antibody responses. A DNA vaccine cocktail for three bunyaviruses (Rift Valley fever virus (RVFV), Crimean Congo hemorrhagic fever virus (CCHFV), and HTNV, and one flavivirus [tick-borne encephalitis virus (TBEV)] was tested in mice alone and in cocktail formulations with immune responses assessed. While the study did not challenge the mice with the pathogens, there were no major differences in the antibody responses between the monovalent and cocktail formulations (103).

A combination DNA vaccine against HTNV and Puumala virus (PUUV), two Old World hantaviruses, was tested and compared to single plasmid DNA vaccines in a four-dose Phase I trial in humans. Neutralizing antibody responses were elicited in 100% of subjects who received the HTNV or PUUV vaccines alone, but only in 44% of subjects who received the combination vaccine against both HTNV and PUUV (105). The difference between the HTNV/PUUV combination and the single formulation could be explained by dosage; the individual formulations used 2 mg each to keep the total amount of DNA delivered consistent, while the combination vaccine group received 1 mg of each plasmid per dose, and it is possible that the lower amount of DNA was not able to generate the same antibody responses as the higher amount (105). However, vaccine interference has also been seen with other HTNV/PUUV combination vaccines and could explain the observed differences. In a Phase 1a clinical trial, when vaccine antigens were combined, more participants responded to the PUUV than to the HTNV DNA vaccine (104, 106). In animal studies, mixing two M-segment DNA vaccines for HTNV and PUUV reduced their immunogenicity in hamsters (106). The mechanism of interference is not understood for these vaccines; however, there is evidence that co-expression of the different hantavirus glycoproteins (Gn and Gc) in cell culture reduces the expression of one or both (106), suggesting there may be competition between the two viral Gn and Gc proteins at the transcriptional or translational level.

Interestingly, a dose-optimizing Phase 2a study using combination PUUV and HTNV DNA vaccines did not show interference or a higher response to either pathogen (107). It was speculated by the authors that codon modification for use in humans, removal of gene elements known to interfere with mammalian expression, and improved mRNA stability from the plasmid for HTNV eliminated the interference seen previously (106). However, single formulation plasmids were not used for comparison, so the promising HTNV and PUUV responses may have been lower when the vaccines were delivered together (107). Surprisingly, the pWRG/HTN-M(x) and pWRG/PUU-M(s2) plasmids used in the Phase 2a study (107) appear to be the same as the plasmids used in the recent Phase I needle-free injection study that showed poor combination outcomes (105). The development of these vaccines and efforts to overcome the interference were reviewed by Schmaljohn et al. in 2014 (108).

ZIKV, Dengue virus serotypes 1-4 (DENV1-4) and CHIKV are arboviruses that co-circulate in regions with *Aedes aegypti* mosquitoes. Three doses of a combination DNA vaccine generated using plasmids expressing ZIKV, DENV 1-4, and CHIKV structural proteins (multiple mosquito-borne viruses (MMBV) vaccine) were injected intramuscularly into mice or intradermally (Mantoux method) in NHPs (101). The co-formulated MMBV vaccine was compared to single formulations of the individual plasmids for each of the DENV strains, ZIKV, and CHIKV. The humoral responses elicited by the cocktail formulation were not significantly different for any virus after three doses, although the DENV responses appeared lower after a single vaccination in the multi-pathogen formulation for DENV 2, DENV 3 and DENV 4 at two weeks post-vaccination (101). However, this was not statistically significant and the range in responses at two weeks post-vaccination in the individual formulation was wide (101). Additionally, for ZIKV, DENV 1, DENV 3, and DENV 4, antibodies were detected in all NHPs vaccinated with the cocktail formulation and in only four out of five in the individual treatment group after two immunizations, a difference that was also not statistically significant but was noted by the authors. There were no significant differences in T cell responses between the cocktail and individual groups for any of the viral antigens at any time point by ELISpot for IFN-γ. Interestingly, there does appear to be a trend at later time points where the DENV 1–4 groups had higher IFN-γ responses after cocktail vaccination compared to individual vaccination. While more animals would be required to determine whether this trend is reproducible, it is plausible that the DENV T cell response to one serotype is stimulated by the other serotypes in the multi-pathogen vaccine due to the sequence similarity of DENV serotypes. Currently, the MMBV vaccine has not been tested with virus challenge in any animal models (101).

A multivalent hemorrhagic fever virus (EBOV, MARV, and LASV) DNA vaccine was tested for immune responses in guinea pigs and NHPs with antibody titers and neutralizing antibodies assessed in both. In guinea pigs, there was a significant decrease in MARV antibodies in response to the multivalent vaccine compared with the monovalent vaccine, and the multivalent vaccine had almost undetectable MARV antibodies. There was no change in the antibodies against EBOV or LASV (101). The authors additionally investigated the delivery of the MARV plasmid with just LASV and just EBOV (bivalent formulations) and showed that combining MARV with either one greatly reduced antibody titers (101). The authors did not proceed with the multivalent formulation of the vaccine in NHPs after the lack of response in guinea pigs. The challenges in achieving good MARV GP expression compared to other filoviruses have been documented *in vitro (*109). However, immunohistochemistry detected the MARV GP in guinea pig skin 24 hours after delivery of the cocktail vaccine, suggesting that poor expression due to competition may not explain the dramatic decrease in anti-MARV responses, and that one of the mechanisms of vaccine interference discussed could play a role.

Unlike DNA vaccines, mRNA vaccines are formulated into lipid nanoparticles (LNP), for delivery into cells. The immune responses elicited by multi-pathogen mRNA vaccines can vary depending on whether the mRNA is formulated into the same LNP, or if multiple LNPs are combined. A multi-pathogen mRNA vaccine against the orthoebolaviruses EBOV, SUDV and BDBV used EBOV, SUDV and BDBV GPs and EBOV nucleoprotein (NP), as antigens and tested the differences in responses when packaged into the same lipid nanoparticle (LNP) ([GPs+NP]@LNP) compared to formulated into separate LNPs (GP@LNPs+NP@LNP) (110). The GP@LNPs+NP@LNP elicited a stronger T cell response, particularly in granzyme B and interferon-γ secretion, compared to the more balanced Th1/Th2 polarization exhibited by [GPs+NP]@LNP (110). NP-specific T cell responses are enhanced in the [GPs+NP]@LNP compared to the separate LNPs, suggesting that the co-expression with the GPs increases the specific T cell responses (110).

## 6 Multi-pathogen filovirus vaccines highlight challenges in assessing vaccine interference

While some multi-pathogen vaccines appear unaffected by cocktail formulations (11), others may have reduced efficacy against one or more pathogens compared to the monovalent equivalent. For example, a statistically significant reduction in anti-MARV immune responses in filovirus cocktail formulations is seen in DNA vaccines (101) and VSV-vector vaccines (62, 63), and reductions in anti-MARV immune responses that were not statistically significant have occurred in some multivalent formulations of Ad vector (51) and protein subunit vaccines (86, 92). It can be difficult to determine whether vaccine interference can be overcome by changing the delivery method or vaccine platform, or whether it is inherent to a specific antigen, because testing is conducted in multiple animal models.

## 7 Comparing mechanisms of vaccine interference across different animal models

Vaccine interference can be specific to the animal species testing occurs in. For example, epitope competition varies across animal models because each expresses different MHC alleles (111). This means that a preference for one T cell epitope may occur in a model and result in vaccine interference, but not exist at all in a different model. Additionally, an outbred animal model may be less vulnerable to extreme dominance by a particular epitope than models homozygous for particular MHC alleles (112), and even the proteolytic processing enzymes necessary to generate peptides for display vary between species (113). The propensity for resource competition in different animal species is more consistent, but there may still be differences in the availability of ribosomes of transcription factors required for antigen expression between species. These factors make it difficult to compare platforms that are tested in different animal models. The multi-filovirus DNA vaccine (101) and Ad vector vaccine (51) both use the same GP antigen sequences (MARV-Angola and EBOV-Mayinga (Yambuku)), but because they were tested in different animal models (guinea pig and cynomolgus macaque NHP models, respectively), any platform-specific differences are obscured.

Additionally, certain animal models have pre-existing biases in immune polarization (114), such as BALB/c mice being predisposed to Th2 polarization (115), which can significantly alter the outcome of vaccination. In the example of the example of ChAdOx1-biEBOV, which protected mice but failed to protect NHPs against SUDV, differences in protection could be due to vaccine interference, but could also be the result of different animal challenges having different stringencies. NHP models of SUDV are a high stringency challenge, particularly in the ChAdOx1-biEBOV example where a high dose of inoculum and the intramuscular.

Finally, codon-optimization can alter DNA and RNA stability which can change the relative efficiency with which two nucleic acids are expressed in different models (116). Comparisons of vaccine platforms are complicated when different animal models are used since model-specific differences may obscure mechanisms of interference. Where possible, using the same animal model can help clarify whether observed effects are platform-related.

## 8 Methods

PubMed, a scholarly repository of journal articles about health and medicine, was searched with the terms in **Figure 2** through July 2025. The list of biodefense-relevant pathogens (and wider viral families) was taken from the CDC bioterrorism agent list (9). The specific search terms are shown in **Figure 2** and were searched in combination, where each of the first terms “Vaccine term” were combined with each of the second terms “Pathogen terms”. This process yielded duplicates (i.e. a Lassa, Marburg and Ebola multi-pathogen vaccine appears three times), which are not removed from the search totals. The search results were manually filtered. Records were removed if they; did not pertain to pathogen vaccines; did not pertain to the pathogens identified by the CDC bioterrorism agent list; or only included vaccination against a single species/virus. Multiple reviewers were involved in the screening process in determining the final set of articles. Additional articles providing relevant mechanisms and broader immunology were searched using PubMed but without systematic screening.

**Figure 2:**
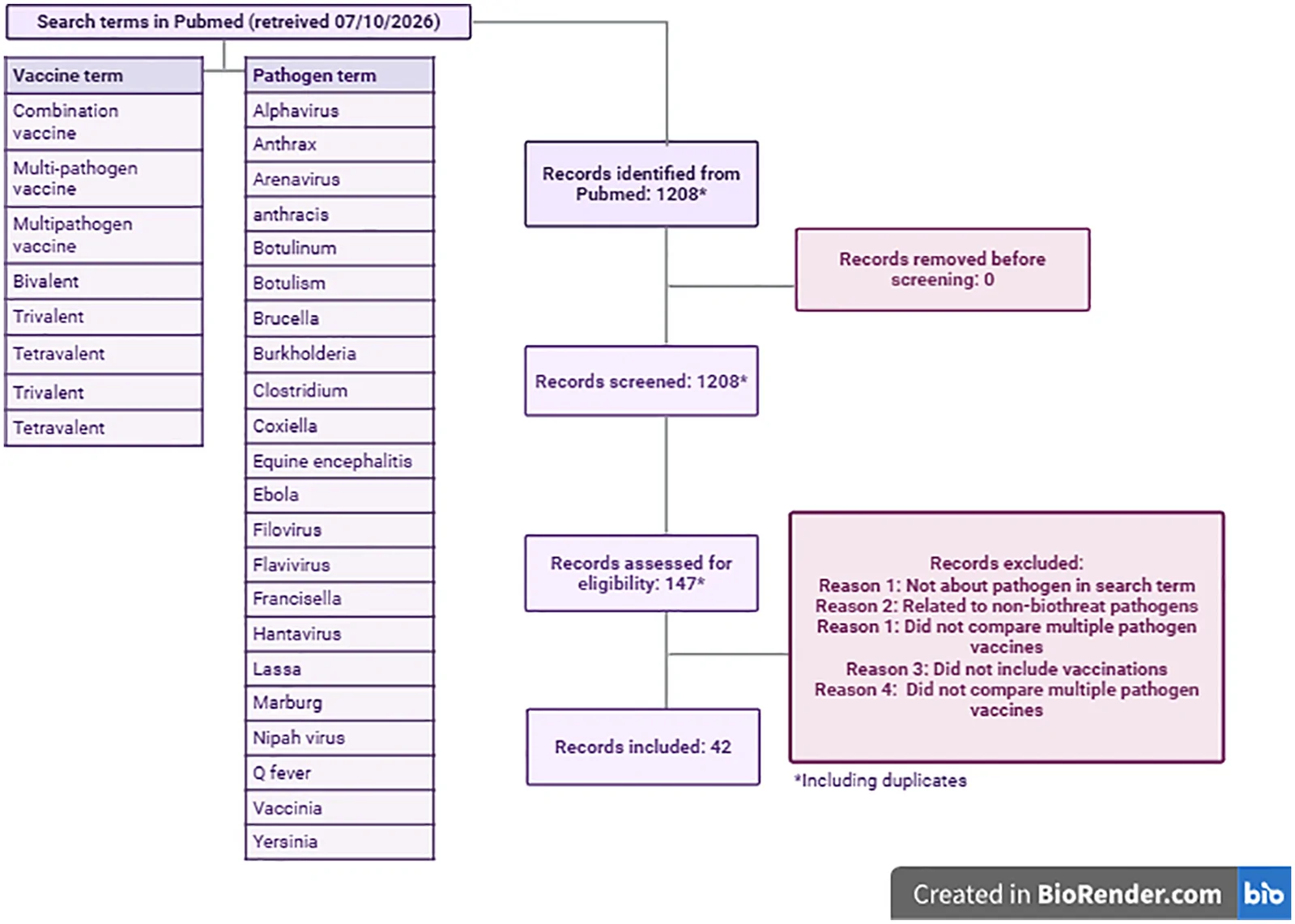
Schematic of search methodology. * indicates duplicates.

## 9 Discussion

It is difficult to predict how vaccines will interact in combination or within multivalent formulations, though some interference mechanisms are platform-specific. **Table 1** groups vaccines by platform to indicate which mechanisms should be considered when assessing co-administered or multi-pathogen vaccines.

Streamlining the development of multi-pathogen vaccines requires attention to three key areas: (1) experimental designs that allow for direct comparison with previous effective or protective single-pathogen vaccines; (2) mechanistic understanding of platform-related challenges; and (3) characterization of immune polarization. Each of these considerations is expanded upon below:

1. Experimental design for comparisons. A practical development sequence should begin with *in vitro* confirmation that each antigen is expressed at the expected level when delivered alone and in combination, followed by optimization of antigen dose and ratio to minimize competition while maintaining protective responses to each pathogen. Candidate formulations should then be evaluated using standardized immune-response assays and predefined, pathogen-appropriate correlates of protection, where available. This staged approach prioritizes relatively rapid antigen-expression and compatibility testing before resource-intensive animal studies, although dose adjustments or platform modifications may increase formulation complexity and may not fully predict immune interactions *in vivo*. Inclusion of monovalent or single-antigen counterparts as controls for multivalent vaccines is essential to enable direct comparison with existing datasets. If experimental constraints limit the number of test groups, standardized assay formats should be used (e.g., normalizing antibody titers to a known monoclonal). These considerations align with broader calls for standardized immunogenicity metrics, harmonized experimental protocols, and transparent reporting to improve comparability across vaccine studies (117). The experimental design should replicate conditions used in single-vaccine experiments including animal age, vaccine dose and volume, challenge details, and timepoints.
2. Platform-specific challenges. Different vaccine platforms and vectors have unique challenges when delivered as combinations. For example, multi-pathogen DNA vaccines may encounter plasmid competition. Similarly, protein expression interference or co-infection limitations in nucleic acid and viral vectored vaccines can be empirically determined with *in vitro* assays that reduce the need for *in vivo* testing of potentially incompatible vaccines.
3. Immune polarization effects. Combining vaccines or antigens can alter cellular immune polarization leading to shifts in antibody isotypes and downstream cellular responses. One proposed mechanism for impaired Hib responses in childhood combination vaccines (Hib and DTaP) involves the difference in adjuvant formulations: Hib vaccination does not contain an adjuvant, and the DTaP formulation contains aluminum salt (110). Although this mechanism is debated (111), vaccine assessment should include polarization metrics relevant to the animal model, such as IgG1/IgG2a ratios in BALB/c mice.

As with single pathogen vaccines, results in one animal model are not guaranteed to translate to another, and immuno-bridging from animal models to human protection remains a significant challenge. For example, the bivalent Ad-vector ChAdOx1-biEBOV protected mice from both EBOV and SUDV challenge but failed to protect NHPs from SUDV challenge despite inducing robust humoral immunity. These findings underscore the importance of evaluating vaccine candidates in multiple models. The predictive value of an animal model depends on the pathogen, the extent to which the challenge reproduces human infection, and whether relevant correlates of protection are known. Species-specific differences in antigen processing, immune polarization, and effector responses, together with differences in challenge strain, dose, route, and study endpoint, can complicate interpretation. Multi-pathogen vaccine studies should therefore assess protection against each component individually and interpret immune responses within the limitations of the selected model.

Multi-pathogen vaccines represent a promising strategy for biodefense research. When appropriately formulated, multi-pathogen vaccines could mitigate the threat posed by multiple biodefense pathogens while serving as a deterrent against the deliberate use of biological agents. In addition, multi-pathogen vaccines could reduce costs, streamline logistics, and shorten timelines for deployment. Careful consideration of vaccine interference will be critical to ensure accurate assessment of vaccine quality and to accelerate the development of effective biodefense vaccines. A unified evaluation framework for multi-pathogen biodefense vaccines should therefore progress from *in vitro* antigen-expression and compatibility testing, to dose and antigen-ratio optimization, and standardized comparison with monovalent controls. Changing the vaccine platform may resolve expression- or vector-specific interference but requires more extensive redevelopment and validation. In an ideal scenario, comparisons across species should use matched antigen amino acid sequences, formulations, doses scaled using a clearly defined rationale, sampling timepoints, challenge conditions, and immune-response assays. Safety assessment should also examine whether subprotective or imbalanced responses increase risks such as antibody-dependent enhancement, enhanced disease, or accelerated mortality following challenge. Applying this framework to vaccines targeting Category A–C biothreat agents would support transparent selection of candidates for stockpiling based on breadth of protection, durability, safety, manufacturability, and readiness for rapid deployment.

## Glossary

Ad: Adenovirus
ANDV: Andes virus
BoNT: Botulinum neurotoxin
CCHFV: Crimean Congo hemorrhagic fever virus
CDC: Centers for disease control
CD4+: Helper T cell
CD8+: Cytotoxic T cell
ChAdOx1: Chimpanzee adenovirus Oxford 1 vector
CHAV: Chapare virus
CHIKV: Chikungunya virus
DENV: Dengue virus
DTaP: Diphtheria, tetanus, and acellular pertussis vaccine
EBOV: Ebola virus
EF: Edema factor
eGFP: Enhanced green fluorescent protein
EVD: Ebola virus disease
GnGc: Glycoproteins
GTOV: Guanarito virus
HCPS: Hantavirus cardiopulmonary syndrome
HFRS: Hemorrhagic fever with renal syndrome
HFV: Hemorrhagic fever viruses
Hib: Hemophilus influenzae type b polysaccharide vaccine
HTNV: Hantaan virus
Ig: Immunoglobulin
JUNV: Junín virus
LASV: Lassa virus
LF: Lethal factor
LVS: *F. tularensis* live vaccine strain
MACV: Machupo virus
MARV: Marburg virus
MHC: Major histocompatibility complex
MMBV: Multiple mosquito borne viruses
MOPV: Mopeia virus
MVA-BN: Modified Vaccinia Ankara-Bavarian Nordic
MVA-filo: Modified Vaccinia Ankara-filo
MVD: Marburg virus disease
NHP: Nonhuman primate
PA: Protective antigen
P_CMV_: Plasmid with cytomegalovirus promoter
PUUV: Puumala virus
RABV: Rabies virus
RVFV: Rift Valley fever virus
rVSV-ZEBOV: recombinant vesicular stomatitis virus-Zaire Ebola virus
SABV: Sabia virus
SARS-CoV-2: Severe acute respiratory syndrome Coronavirus 2
SCV: Sementis Copenhagen Vector
SUDV: Sudan virus
TAFV: Taï Forest virus
TBEV: Tick-borne encephalitis virus
TCR: T cell receptor
Th1: T helper 1 response
Th2: T helper 2 response
VEEV: Venezuelan equine encephalitis virus
VLP: Virus-like particle
VSV: Vesicular stomatitis virus
WNV: West Nile virus
YFV: Yellow Fever virus

## References

[1] Shang Y Li H Zhang R . Effects of pandemic outbreak on economies: evidence from business history context. Front Public Health. (2021) 9:632043. doi: 10.3389/fpubh.2021.632043 33777885 PMC7994505

[2] Poland GA Jacobson RM Tilburt J Nichol K . The social, political, ethical, and economic aspects of biodefense vaccines. Vaccine. (2009) 27:D23–7. doi: 10.1016/j.vaccine.2009.08.054 19837281 PMC2831650

[3] Domínguez-Andrés J van Crevel R Divangahi M Netea MG . Designing the next generation of vaccines: relevance for future pandemics. MBio. (2020) 11. doi: 10.1128/mbio.02616-20 PMC8534290 33443120

[4] Skibinski DA Baudner BC Singh M O’Hagan DT . Combination vaccines. J Global Infect Dis. (2011) 3:63–72. doi: 10.4103/0974-777x.77298 21572611 PMC3068581

[5] Lu S Wang S Grimes-Serrano JM . Current progress of DNA vaccine studies in humans. Expert Rev Vaccines. (2008) 7:175–91. doi: 10.1586/14760584.7.2.175 18324888

[6] Beaumier CM Gomez-Rubio AM Hotez PJ Weina PJ . United States military tropical medicine: extraordinary legacy, uncertain future. PLoS NeglTrop Dis. (2013) 7:e2448. doi: 10.1371/journal.pntd.0002448 24386494 PMC3873258

[7] Pita R Gunaratna R . Anthrax as a biological weapon: from World War I to the Amerithrax investigation. Int J Intell Counterintelligence. (2010) 23:61–103.

[8] Renault V Humblet M-F Saegerman C . Biosecurity concept: Origins, evolution and perspectives. Animals. (2021) 12:63. doi: 10.3390/ani12010063 35011169 PMC8749630

[9] Sandrock C . 40 - bioterrorism. In: Broaddus VC Mason RJ Ernst JD King TE Lazarus SC Murray JF , editors. Murray and Nadel's Textbook of Respiratory Medicine (Sixth Edition). W.B. Saunders, Philadelphia (2016). p. 699–712.e2.

[10] Herder M Graham JE Gold R . From discovery to delivery: public sector development of the rVSV-ZEBOV Ebola vaccine. J Law Biosci. (2020) 7. doi: 10.1093/jlb/lsz019 34221434 PMC8249092

[11] Lauer KB Borrow R Blanchard TJ . Multivalent and multipathogen viral vector vaccines. Clin Vaccine Immunol. (2017) 24. doi: 10.1128/cvi.00298-16 27535837 PMC5216423

[12] Vidor E . The nature and consequences of intra-and inter-vaccine interference. J Comp Pathol. (2007) 137:S62–6. doi: 10.1016/j.jcpa.2007.04.014 17560593

[13] Akram A Inman RD . Immunodominance: a pivotal principle in host response to viral infections. Clin Immunol. (2012) 143:99–115. doi: 10.1016/j.clim.2012.01.015 22391152

[14] Shu J Shen W Liu H Zhou Y Li J Zhuang Y . The immunologic dominance of an epitope within a rationally designed poly-epitope vaccine is influenced by multiple factors. Vaccine. (2020) 38:2913–24. doi: 10.1016/j.vaccine.2020.02.069 32127225

[15] Kim CG Lee JC Ju DB Kim SK Yun CH Cho CS . Enhancement of immune responses elicited by nanovaccines through a cross-presentation pathway. Tissue Eng Regener Med. (2023) 20:355–70. doi: 10.1007/s13770-023-00527-y 36884197 PMC9994410

[16] Lee W Suresh M . Vaccine adjuvants to engage the cross-presentation pathway. Front Immunol. (2022) 13:940047. doi: 10.3389/fimmu.2022.940047 35979365 PMC9376467

[17] Kim A Sadegh-Nasseri S . Determinants of immunodominance for CD4 T cells. Curr Opin Immunol. (2015) 34:9–15. doi: 10.1016/j.coi.2014.12.005 25576665 PMC4444386

[18] Weaver JM Lazarski CA Richards KA Chaves FA Jenks SA Menges PR . Immunodominance of CD4 T cells to foreign antigens is peptide intrinsic and independent of molecular context: implications for vaccine design. J Immunol. (2008) 181:3039–48. doi: 10.4049/jimmunol.181.5.3039 18713974 PMC2814425

[19] Kim A Boronina TN Cole RN Darrah E Sadegh-Nasseri S . Distorted immunodominance by linker sequences or other epitopes from a second protein antigen during antigen-processing. Sci Rep. (2017) 7:46418. doi: 10.1038/srep46418 28422163 PMC5396073

[20] Jenkins MR Webby R Doherty PC Turner SJ . Addition of a prominent epitope affects influenza A virus-specific CD8+ T cell immunodominance hierarchies when antigen is limiting. J Immunol. (2006) 177:2917–25. doi: 10.4049/jimmunol.177.5.2917 16920927

[21] Cassotta A Paparoditis P Geiger R Mettu RR Landry SJ Donati A . Deciphering and predicting CD4+ T cell immunodominance of influenza virus hemagglutinin. J Exp Med. (2020) 217:e20200206. doi: 10.1084/jem.20200206 32644114 PMC7537397

[22] Nestić D Božinović K Pehar I Wallace R Parker AL Majhen D . The revolving door of adenovirus cell entry: not all pathways are equal. Pharmaceutics. (2021) 13. 10.3390/pharmaceutics13101585 PMC8540258 34683878

[23] Angeletti D Yewdell JW . Understanding and manipulating viral immunity: antibody immunodominance enters center stage. Trends Immunol. (2018) 39:549–61. doi: 10.1016/j.it.2018.04.008 29789196

[24] Angeletti D Gibbs JS Angel M Kosik I Hickman HD Frank GM . Defining B cell immunodominance to viruses. Nat Immunol. (2017) 18:456–63. doi: 10.1038/ni.3680 28192417 PMC5360521

[25] Pavel AB Glickman JW Michels JR Kim-Schulze S Miller RL Guttman-Yassky E . Th2/Th1 cytokine imbalance is associated with higher COVID-19 risk mortality. Front Genet. (2021) 12:706902. doi: 10.3389/fgene.2021.706902 34335703 PMC8324177

[26] André FE . Development and clinical application of new polyvalent combined paediatric vaccines. Vaccine. (1999) 17:1620–7. 10.1016/s0264-410x(98)00426-5 10194814

[27] Eskola J Ölander R-M Hovi T Litmanen L Peltola S Käyhty H . Randomised trial of the effect of co-administration with acellular pertussis DTP vaccine on immunogenicity of Haemophilus influenzae type b conjugate vaccine. Lancet. (1996) 348:1688–92. doi: 10.1016/s0140-6736(96)04356-5 8973430

[28] Mawas F Dickinson R Douglas-Bardsley A Xing DKL Sesardic D Corbel MJ . Immune interaction between components of acellular pertussis-diphtheria-tetanus (DTaP) vaccine and Haemophilus influenzae b (Hib) conjugate vaccine in a rat model. Vaccine. (2006) 24:3505–12. doi: 10.1016/j.vaccine.2006.02.021 16524648

[29] Rowe J Yerkovich S Richmond P Suriyaarachchi D Fisher E Feddema L . Th2-associated local reactions to the acellular diphtheria-tetanus-pertussis vaccine in 4- to 6-year-old children. Infect Immun. (2005) 73:8130–5. doi: 10.1128/iai.73.12.8130-8135.2005 16299307 PMC1307058

[30] Smith WJ Thompson R Egan PM Zhang Y Indrawati L Skinner JM . Impact of aluminum adjuvants on the stability of pneumococcal polysaccharide-protein conjugate vaccines. Vaccine. (2023) 41:5113–25. doi: 10.1016/j.vaccine.2023.05.059 37321893

[31] Grifantini R Finco O Bartolini E Draghi M Del Giudice G Kocken C . Multi-plasmid DNA vaccination avoids antigenic competition and enhances immunogenicity of a poorly immunogenic plasmid. Eur J Immunol. (1998) 28:1225–32. doi: 10.1002/(sici)1521-4141(199804)28:04<1225::aid-immu1225>3.0.co;2-6 9565362

[32] Warfield KL Swenson DL Negley DL Schmaljohn AL Aman MJ Bavari S . Marburg virus-like particles protect guinea pigs from lethal Marburg virus infection. Vaccine. (2004) 22:3495–502. doi: 10.1016/j.vaccine.2004.01.063 15308377

[33] Anywaine Z Whitworth H Kaleebu P Praygod G Shukarev G Manno D . Safety and immunogenicity of a 2-dose heterologous vaccination regimen with Ad26. ZEBOV and MVA-BN-Filo Ebola vaccines: 12-month data from a phase 1 randomized clinical trial in Uganda and Tanzania. J Infect Dis. (2019) 220:46–56. doi: 10.1093/infdis/jiz070 30796818 PMC6548900

[34] Jo S Li L Thakur C Telfer KA Sultan H Ohara RA . mRNA vaccines engage unconventional pathways in CD8(+) T cell priming. Nature. (2026) 654:485–94. doi: 10.1038/s41586-026-10353-6 41986715 PMC13089314

[35] Mawas F Newman G Burns S Corbel MJ . Suppression and modulation of cellular and humoral immune responses to Haemophilus influenzae type B (Hib) conjugate vaccine in hib-diphtheria-tetanus toxoids-acellular pertussis combination vaccines: a study in a rat model. J Infect Dis. (2005) 191:58–64. 10.1086/426396 15593004

[36] Di Blasi R Marbiah MM Siciliano V Polizzi K Ceroni F . A call for caution in analysing mammalian co-transfection experiments and implications of resource competition in data misinterpretation. Nat Commun. (2021) 12:2545. doi: 10.1038/s41467-021-22795-9 33953169 PMC8099865

[37] Huliák I Sike Á Zencir S Boros IM . The objectivity of reporters: interference between physically unlinked promoters affects reporter gene expression in transient transfection experiments. DNA Cell Biol. (2012) 31:1580–7. 10.1089/dna.2012.1711 22994211

[38] Frei T Cella F Tedeschi F Gutiérrez J Stan G-B Khammash M . Characterization and mitigation of gene expression burden in mammalian cells. Nat Commun. (2020) 11:4641. doi: 10.1038/s41467-020-18392-x 32934213 PMC7492461

[39] Jain A Margaliot M Gupta AK . Large-scale mRNA translation and the intricate effects of competition for the finite pool of ribosomes. J R Soc Interface. (2022) 19:20220033. doi: 10.1098/rsif.2022.0033 35259953 PMC8922411

[40] Raveh A Margaliot M Sontag ED Tuller T . A model for competition for ribosomes in the cell. J R Soc Interface. (2016) 13. doi: 10.1098/rsif.2015.1062 26962028 PMC4843671

[41] Sokabe M Fraser CS . It’s a competitive business. eLife. (2024) 13:e96304. 10.7554/eLife.96304 PMC10890784 38393777

[42] Zhou F Bocetti JM Hou M Qin D Hinnebusch AG Lorsch JR . Transcriptome-wide analysis of the function of Ded1 in translation preinitiation complex assembly in a reconstituted *in vitro* system. eLife. (2024) 13:RP93255. doi: 10.7554/elife.93255 38573742 PMC10994665

[43] Bessonov N Neverova D Popov V Volpert V . Emergence and competition of virus variants in respiratory viral infections. Front Immunol. (2022) 13:945228. 10.3389/fimmu.2022.945228 PMC10165551 37168105

[44] Shattock AJ Johnson HC Sim SY Carter A Lambach P Hutubessy RC . Contribution of vaccination to improved survival and health: modelling 50 years of the Expanded Programme on Immunization. Lancet. (2024) 403:2307–16. doi: 10.1016/s0140-6736(24)00850-x 38705159 PMC11140691

[45] Guzman MG Vazquez S . The complexity of antibody-dependent enhancement of dengue virus infection. Viruses. (2010) 2:2649–62. doi: 10.3390/v2122649 21994635 PMC3185591

[46] Swenson DL Warfield KL Negley DL Schmaljohn A Aman MJ Bavari S . Virus-like particles exhibit potential as a pan-filovirus vaccine for both Ebola and Marburg viral infections. Vaccine. (2005) 23:3033–42. doi: 10.1016/j.vaccine.2004.11.070 15811650

[47] Warfield KL Bosio CM Welcher BC Deal EM Mohamadzadeh M Schmaljohn A . Ebola virus-like particles protect from lethal Ebola virus infection. Proc Natl Acad Sci U S A. (2003) 100:15889–94. doi: 10.1073/pnas.2237038100 14673108 PMC307663

[48] Bliss-Moreau E Amara RR Buffalo EA Colman RJ Embers ME Morrison JH . Improving rigor and reproducibility in nonhuman primate research. Am J Primatol. (2021) 83:e23331. doi: 10.1002/ajp.23331 34541703 PMC8629848

[49] Charan J Kantharia N . How to calculate sample size in animal studies? J Pharmacol Pharmacotherapeutics. (2013) 4:303–6. doi: 10.4103/0976-500x.119726 24250214 PMC3826013

[50] Chavda VP Bezbaruah R Valu D Patel B Kumar A Prasad S . Adenoviral vector-based vaccine platform for COVID-19: current status. Vaccines. (2023) 11:432. doi: 10.3390/vaccines11020432 36851309 PMC9965371

[51] Callendret B Vellinga J Wunderlich K Rodriguez A Steigerwald R Dirmeier U . A prophylactic multivalent vaccine against different filovirus species is immunogenic and provides protection from lethal infections with Eb olavirus and Marburgvirus species in non-human primates. PLoS One. (2018) 13:e0192312. doi: 10.1371/journal.pone.0192312 29462200 PMC5819775

[52] Bockstal V Shukarev G McLean C Goldstein N Bart S Gaddah A . First-in-human study to evaluate safety, tolerability, and immunogenic ity of heterologous regimens using the multivalent filovirus vaccines Ad26.Filo and MVA-BN-Filo administered in different sequences and sche dules: A randomized, controlled study. PLoS One. (2022) 17:e0274906. doi: 10.1371/journal.pone.0274906 36197845 PMC9534391

[53] Flaxman A Sebastian S Appelberg S Cha KM Ulaszewska M Purushotham J . Potent immunogenicity and protective efficacy of a multi-pathogen vacc ination targeting Ebola, Sudan, Marburg and Lassa viruse. PLoS Pathog. (2024) 20:e1012262. doi: 10.1371/journal.ppat.1012262 38924060 PMC11233014

[54] van Tol S Fletcher P Feldmann F Mukesh RK Port JR Gallogly S . A bivalent adenovirus-vectored vaccine induces a robust humoral response, but does not protect cynomolgus macaques against a lethal challenge with Sudan virus. J Infect Dis. (2024) 230:1083–92. doi: 10.1093/infdis/jiae056 38487996 PMC11566226

[55] Pratt WD Wang D Nichols DK Luo M Woraratanadharm J Dye JM . Protection of nonhuman primates against two species of Ebola virus infection with a single complex adenovirus vector. Clin Vaccine Immunol. (2010) 17:572–81. doi: 10.1128/cvi.00467-09 20181765 PMC2849326

[56] Sebastian S Flaxman A Cha KM Ulaszewska M Gilbride C Sharpe H . A multi-filovirus vaccine candidate: Co-expression of Ebola, Sudan, an d Marburg antigens in a single vector. Vaccines. (2020) 8:241. doi: 10.3390/vaccines8020241 32455764 PMC7349952

[57] Perdiguero B Pérez P Marcos-Villar L Albericio G Astorgano D Álvarez E . Highly attenuated poxvirus-based vaccines against emerging viral disea ses. J Mol Biol. (2023) 435:168173. doi: 10.1016/j.jmb.2023.168173 37301278 PMC10249371

[58] Choi E-L Lacarra B Afolabi MO Ale BM Baiden F Bétard C . Safety and immunogenicity of the two-dose heterologous Ad26.ZEBOV and MVA-BN-Filo Ebola vaccine regimen in infants: a phase 2, randomised, d ouble-blind, active-controlled trial in Guinea and Sierra Leone. Lancet Global Health. (2023) 11:e1743–52. doi: 10.1016/s2214-109x(23)00410-2 37858585

[59] Pfranger M Krause N Asbach B Meier J Carnell G Scheer L . Immunogenicity of a trivalent haemorrhagic fever vaccine candidate against Sudan virus, Marburg virus and Lassa virus in an mpox vaccine. J Gen Virol. (2025) 106. doi: 10.1099/jgv.0.002157 41051941 PMC12500382

[60] Tiemessen MM Solforosi L Dekking L Czapska-Casey D Serroyen J Sullivan NJ . Protection against Marburg virus and Sudan virus in NHP by an adenovec tor-based trivalent vaccine regimen is correlated to humoral immune re sponse levels. Vaccines. (2022) 10:1263. doi: 10.3390/vaccines10081263 36016151 PMC9412258

[61] Woolsey C Strampe J Fenton KA Agans KN Martinez J Borisevich V . A recombinant vesicular stomatitis virus-based vaccine provides postexposure protection against Bundibugyo ebolavirus infection. J Infect Dis. (2023) 228:S712–s720. doi: 10.1093/infdis/jiad207 37290053 PMC10651203

[62] Matassov D Mire CE Latham T Geisbert JB Xu R Ota-Setlik A . Single-dose trivalent VesiculoVax vaccine protects macaques from lethal Ebolavirus and Marburgvirus challenge. J Virol. (2018) 92. doi: 10.1128/jvi.01190-17 PMC5774882 29142131

[63] Cross RW Xu R Matassov D Hamm S Latham TE Gerardi CS . Quadrivalent VesiculoVax vaccine protects nonhuman primates from viral-induced hemorrhagic fever and death. J Clin Invest. (2020) 130:539–51. 10.1172/JCI131958 PMC6934204 31820871

[64] Kurup D Fisher CR Scher G Yankowski C Testa A Keshwara R . Tetravalent rabies-vectored filovirus and Lassa fever vaccine induces long-term immunity in nonhuman primates. J Infect Dis. (2021) 224:995–1004. doi: 10.1093/infdis/jiab014 33421072 PMC8448432

[65] Chattopadhyay A Aguilar PV Bopp NE Yarovinsky TO Weaver SC Rose JK . A recombinant virus vaccine that protects against both Chikungunya and Zika virus infections. Vaccine. (2018) 36:3894–900. doi: 10.1016/j.vaccine.2018.05.095 29807712 PMC13224337

[66] Scher G Schnell MJ . Rhabdoviruses as vectors for vaccines and therapeutics. Curr Opin Virol. (2020) 44:169–82. doi: 10.1016/j.coviro.2020.09.003 33130500 PMC8331071

[67] Cross RW Woolsey C Prasad AN Borisevich V Agans KN Deer DJ . A recombinant VSV-vectored vaccine rapidly protects nonhuman primates against heterologous lethal Lassa fever. Cell Rep. (2022) 40:111094. doi: 10.1016/j.celrep.2022.111094 35858566

[68] Scher G Yankowski C Kurup D Josleyn NM Wilkinson ER Wells J . Inactivated rabies-based Lassa fever virus vaccine candidate LASSARAB protects nonhuman primates from lethal disease. NPJ Vaccines. (2024) 9:143. doi: 10.1038/s41541-024-00930-z 39122759 PMC11315998

[69] Emanuel J Callison J Dowd KA Pierson TC Feldmann H Marzi A . A VSV-based Zika virus vaccine protects mice from lethal challenge. Sci Rep. (2018) 8:11043. doi: 10.1038/s41598-018-29401-x 30038228 PMC6056530

[70] Prow NA Liu L Nakayama E Cooper TH Yan K Eldi P . A vaccinia-based single vector construct multi-pathogen vaccine protec ts against both Zika and chikungunya viruses. Nat Commun. (2018) 9:1230. doi: 10.1038/s41467-018-03662-6 29581442 PMC5964325

[71] Prow NA Liu L McCarthy MK Walters K Kalkeri R Geiger J . The vaccinia virus based Sementis Copenhagen Vector vaccine against Zika and chikungunya is immunogenic in non-human primates. NPJ Vaccines. (2020) 5:44. doi: 10.1038/s41541-020-0191-8 32550013 PMC7265471

[72] Reynard S Carnec X Picard C Borges-Cardoso V Journeaux A Mateo M . A MOPEVAC multivalent vaccine induces sterile protection against New W orld arenaviruses in non-human primates. Nat Microbiol. (2023) 8:64–76. doi: 10.1038/s41564-022-01281-y 36604507

[73] Gao N Yang T Dong L Tang W Cao K Ding L . A multi-antigen vaccinia vaccine broadly protected mice against SARS-CoV-2 and influenza A virus while also targeting SARS-CoV-1 and MERS-CoV. Front Immunol. (2024) 15:1473428. doi: 10.3389/fimmu.2024.1473428 39669563 PMC11634893

[74] Park DB Ahn BE Son H Lee YR Kim YR Jo SK . Construction of a bivalent vaccine against anthrax and smallpox using the attenuated vaccinia virus KVAC103. BMC Microbiol. (2021) 21:76. doi: 10.1186/s12866-021-02121-5 33685392 PMC7938549

[75] Prow NA Jimenez Martinez R Hayball JD Howley PM Suhrbier A . Poxvirus-based vector systems and the potential for multi-valent and multi-pathogen vaccines. Expert Rev Vaccines. (2018) 17:925–34. doi: 10.1080/14760584.2018.1522255 30300041

[76] Jia Q Bowen R Dillon BJ Masleša-Galić S Chang BT Kaidi AC . Single vector platform vaccine protects against lethal respiratory challenge with Tier 1 select agents of anthrax, plague, and tularemia. Sci Rep. (2018) 8:7009. doi: 10.1038/s41598-018-24581-y 29725025 PMC5934503

[77] Tullius MV Bowen RA Back PS Masleša-Galić S Nava S Horwitz MA . LVS ΔcapB-vectored multiantigenic melioidosis vaccines protect against lethal respiratory Burkholderia pseudomallei challenge in highly sensitive BALB/c mice. mBio. (2024) 15:e0018624. doi: 10.1128/mbio.00186-24 38511933 PMC11005352

[78] Jia Q Lee B-Y Bowen R Dillon BJ Som SM Horwitz MA . A *Francisella tularensis* live vaccine strain (LVS) mutant with a deletion in *capB*, encoding a putative capsular biosynthesis protein, is significantly more attenuated than LVS yet induces potent protective immunity in mice against *F. tularensis* challenge. Infect Immun. (2010) 78:4341–55. doi: 10.1128/iai.00192-10 20643859 PMC2950357

[79] Jia Q Bowen RA Masleša-Galić S Horwitz MA . rLVS ΔcapB/Yp F1-V single vector platform vaccine expressing Yersinia pestis F1 and LcrV antigens provides complete protection against lethal respiratory challenge with virulent plague bacilli. Hum Vaccin Immunother. (2025) 21:2507475. doi: 10.1080/21645515.2025.2507475 40417828 PMC12118393

[80] Lam LKM Gilliland T Jr. Dunn M Alcorn-Burckhardt MD Terada Y Sun C . An inactivated trivalent virion-based vaccine protects against aerosol challenge with encephalitic alphaviruses in mice and macaques. Sci Transl Med. (2025) 17:eadv7079. doi: 10.1126/scitranslmed.adv7079 41032627 PMC13019386

[81] Zhu Y He W Hu R Liu X Li M Liu Y . Sindbis virus replicon-based SARS-CoV-2 and dengue combined vaccine candidates elicit immune responses and provide protective immunity in mice. Vaccines (Basel). (2024) 12. doi: 10.3390/vaccines12111292 39591194 PMC11599113

[82] Schmitz KS Comvalius AD Nieuwkoop NJ Geers D Weiskopf D Ramsauer K . A measles virus-based vaccine induces robust chikungunya virus-specific CD4(+) T-cell responses in a phase II clinical trial. Vaccine. (2023) 41:6495–504. doi: 10.1016/j.vaccine.2023.09.022 37726181

[83] Zhou J Zhang X Yao Y Xie Y Li F Wang W . Recombinant live attenuated measles virus-based vaccines inducing comprehensive protection against Ebola and Marburg viruses. Mol Ther. (2025) 33:5470–88. doi: 10.1016/j.ymthe.2025.08.022 40836428 PMC12628153

[84] Zuniga A Wang Z Liniger M Hangartner L Caballero M Pavlovic J . Attenuated measles virus as a vaccine vector. Vaccine. (2007) 25:2974–83. doi: 10.1016/j.vaccine.2007.01.064 17303293 PMC3707277

[85] Georgopoulos AP James LM . Anthrax vaccines in the 21st century. Vaccines. (2024) 12:159. doi: 10.3390/vaccines12020159 38400142 PMC10892718

[86] Lehrer AT Chuang E Namekar M Williams CA Wong TAS Lieberman MM . Recombinant protein filovirus vaccines protect cynomolgus macaques fro m Ebola, Sudan, and Marburg viruses. Front Immunol. (2021) 12:703986. doi: 10.3389/fimmu.2021.703986 34484200 PMC8416446

[87] Morefield GL Tammariello RF Purcell BK Worsham PL Chapman J Smith LA . An alternative approach to combination vaccines: intradermal administration of isolated components for control of anthrax, botulism, plague and staphylococcal toxic shock. J Immune Based Ther Vaccines. (2008) 6:5. doi: 10.1186/1476-8518-6-5 18768085 PMC2543000

[88] Vance DJ Rong Y Brey RN Mantis NJ . Combination of two candidate subunit vaccine antigens elicits protective immunity to ricin and anthrax toxin in mice. Vaccine. (2015) 33:417–21. doi: 10.1016/j.vaccine.2014.11.036 25475957 PMC4274239

[89] Tao P Mahalingam M Zhu J Moayeri M Kirtley ML Fitts EC . A bivalent anthrax-plague vaccine that can protect against two Tier-1 bioterror pathogens, Bacillus anthracis and Yersinia pestis. Front Immunol. (2017) 8:687. doi: 10.3389/fimmu.2017.00687 28694806 PMC5483451

[90] Baldwin MR Tepp WH Przedpelski A Pier CL Bradshaw M Johnson EA . Subunit vaccine against the seven serotypes of botulism. Infect Immun. (2008) 76:1314–8. doi: 10.1128/iai.01025-07 18070903 PMC2258812

[91] Felgner J Clarke E Hernandez-Davies JE Jan S Wirchnianski AS Jain A . Broad antibody and T cell responses to Ebola, Sudan, and Bundibugyo ebolaviruses using mono- and multi-valent adjuvanted glycoprotein vaccines. Antiviral Res. (2024) 225:105851. doi: 10.1016/j.antiviral.2024.105851 38458540 PMC12513533

[92] Lehrer AT Wong T-A Lieberman MM Johns L Medina L Feldmann F . Recombinant subunit vaccines protect guinea pigs from lethal Ebola vir us challenge. Vaccine. (2019) 37:6942–50. doi: 10.1016/j.vaccine.2019.06.035 31324500 PMC6825541

[93] Lehrer AT Wong T-A Lieberman MM Humphreys T Clements DE Bakken RR . Recombinant proteins of Zaire ebolavirus induce potent humoral and cellular immune responses and protect against live virus infection in mice. Vaccine. (2018) 36:3090–100. doi: 10.1016/j.vaccine.2017.01.068 28216187 PMC8426131

[94] Mayerlen LI Wong TAS To A Smith O Chuang E Donini O . Thermostable bivalent & trivalent filovirus vaccines from insect cells: potency demonstrated after 3 months and 2 years. Vaccine. (2025) 64:127684. doi: 10.2139/ssrn.5142338 PMC12479189 40902221

[95] Walpita P Cong Y Jahrling PB Rojas O Postnikova E Yu S . A VLP-based vaccine provides complete protection against Nipah virus challenge following multiple-dose or single-dose vaccination schedules in a hamster model. NPJ Vaccines. (2017) 2:21. doi: 10.1038/s41541-017-0023-7 29263876 PMC5627259

[96] Ithinji DG Buchholz DW Ezzatpour S Monreal IA Cong Y Sahler J . Multivalent viral particles elicit safe and efficient immunoprotection against Nipah Hendra and Ebola viruses. NPJ Vaccines. (2022) 7:166. doi: 10.1038/s41541-022-00588-5 36528644 PMC9759047

[97] Li Q Huang WC Mahmoud SH Ye C Sia ZR Song Y . A subunit vaccine for multiple respiratory viruses. Sci Adv. (2026) 12:eaea3227. doi: 10.1126/sciadv.aea3227 42361166 PMC13308616

[98] Lovell JF Miura K Baik YO Lee C Choi Y Her H . Interim safety and immunogenicity analysis of the EuCorVac-19 COVID-19 vaccine in a Phase 3 randomized, observer-blind, immunobridging trial in the Philippines. J Med Virol. (2024) 96:e29927. doi: 10.1002/jmv.29927 39318203

[99] Ruckwardt TJ Morabito KM Graham BS . Immunological lessons from respiratory syncytial virus vaccine development. Immunity. (2019) 51:429–42. doi: 10.1016/j.immuni.2019.08.007 31533056

[100] Kim NY Son WR Lee MH Choi HS Choi JY Song YJ . A multipathogen DNA vaccine elicits protective immune responses against two class A bioterrorism agents, anthrax and botulism. Appl Microbiol Biotechnol. (2022) 106:1531–42. doi: 10.1007/s00253-022-11812-6 35141866 PMC8979915

[101] Jiang J Ramos SJ Bangalore P Elwood D Cashman KA Kudchodkar SB . Multivalent DNA vaccines as a strategy to combat multiple concurrent e pidemics: Mosquito-borne and hemorrhagic fever viruses. Viruses. (2021) 13:382. doi: 10.3390/v13030382 33673603 PMC7997291

[102] Riemenschneider J Garrison A Geisbert J Jahrling P Hevey M Negley D . Comparison of individual and combination DNA vaccines for B. anthracis, Ebola virus, Marburg virus and Venezuelan equine encephalitis virus. Vaccine. (2003) 21:4071–80. doi: 10.1016/s0264-410x(03)00362-1 12922144

[103] Spik K Shurtleff A McElroy AK Guttieri MC Hooper JW Schmaljohn C . Immunogenicity of combination DNA vaccines for Rift Valley fever virus , tick-borne encephalitis virus, Hantaan virus, and Crimean Congo hemo rrhagic fever virus. Vaccine. (2006) 24:4657–66. doi: 10.1016/j.vaccine.2005.08.034 16174542

[104] Spik KW Badger C Mathiessen I Tjelle T Hooper JW Schmaljohn C . Mixing of M segment DNA vaccines to Hantaan virus and Puumala virus re duces their immunogenicity in hamsters. Vaccine. (2008) 26:5177–81. doi: 10.1016/j.vaccine.2008.03.097 18482782

[105] Hooper JW Kwilas SA Josleyn M Norris S Hutter JN Hamer M . Phase 1 clinical trial of Hantaan and Puumala virus DNA vaccines deliv ered by needle-free injection. NPJ Vaccines. (2024) 9:1–13. doi: 10.1038/s41541-024-00998-7 39551791 PMC11570633

[106] Hooper JW Moon JE Paolino KM Newcomer R McLain DE Josleyn M . A phase 1 clinical trial of Hantaan virus and Puumala virus M-segment DNA vaccines for haemorrhagic fever with renal syndrome delivered by i ntramuscular electroporation. Clin Microbiol Infection. (2014) 20:110–7. doi: 10.1111/1469-0691.12553 24447183

[107] Hooper J Paolino KM Mills K Kwilas S Josleyn M Cohen M . A phase 2a randomized, double-blind, dose-optimizing study to evaluate the immunogenicity and safety of a bivalent DNA vaccine for hemorrhag ic fever with renal syndrome delivered by intramuscular electroporatio n. Vaccines. (2020) 8:377. doi: 10.3390/vaccines8030377 32664486 PMC7565952

[108] Schmaljohn CS Spik KW Hooper JW . DNA vaccines for HFRS: Laboratory and clinical studies. Virus Res. (2014) 187:91–6. doi: 10.1016/j.virusres.2013.12.020 24370868

[109] Clarke EC Collar AL Ye C Caì Y Anaya E Rinaldi D . Production and purification of filovirus glycoproteins in insect and mammalian cell lines. Sci Rep. (2017) 7:15091. doi: 10.1038/s41598-017-15416-3 29118454 PMC5678155

[110] Zhang J Zhang X Yao Y Zhou J Chen D Shen Y . Multivalent mRNA vaccine platform with compatible antigens conferred broad-spectrum protection against orthoebolaviruses' exposure. Proc Natl Acad Sci U S A. (2026) 123:e2517814123. doi: 10.1073/pnas.2517814123 42150061 PMC13213939

[111] Tadros DM Racle J Gfeller D . Predicting MHC-I ligands across alleles and species: How far can we go? Genome Med. (2025) 17:25. doi: 10.1186/s13073-025-01450-8 40114147 PMC11927126

[112] Shkurnikov MY Tonevitskaya SA Stepanova EV Slobodov SA . Immunocompetent mice as a model for preclinical studies of mRNA vaccine immunogenicity. Dokl Biochem Biophys. (2023) 512:266–9. doi: 10.1134/s160767292370045x 38093128 PMC10719129

[113] Blum JS Wearsch PA Cresswell P . Pathways of antigen processing. Annu Rev Immunol. (2013) 31:443–73. doi: 10.1146/annurev-immunol-032712-095910 23298205 PMC4026165

[114] Liu T Matsuguchi T Tsuboi N Yajima T Yoshikai Y . Differences in expression of toll-like receptors and their reactivities in dendritic cells in BALB/c and C57BL/6 mice. Infect Immun. (2002) 70:6638–45. doi: 10.1128/iai.70.12.6638-6645.2002 12438336 PMC133006

[115] Watanabe H Numata K Ito T Takagi K Matsukawa A . Innate immune response in Th1- and Th2-dominant mouse strains. Shock. (2004) 22:460–66. doi: 10.1097/01.shk.0000142249.08135.e9 15489639

[116] Presnyak V Alhusaini N Chen YH Martin S Morris N Kline N . Codon optimality is a major determinant of mRNA stability. Cell. (2015) 160:1111–24. doi: 10.1016/j.cell.2015.02.029 25768907 PMC4359748

[117] Kubicek-Sutherland JZ McMahon B Skowronski E Webb RP Pals T . Editorial: Towards the rapid and systematic assessment of vaccine technologies. Front Immunol. (2026) 17:1816160. doi: 10.3389/fimmu.2026.1816160 41884854 PMC13010855

