# Supplementary material for: Multi-pathogen and combination vaccines for biodefense pathogens
Source: Front Immunol. 2026 Oct 16;17:1945700. doi: 10.3389/fimmu.2026.1945700 (PMC13720360; doi:10.3389/fimmu.2026.1945700)
Supplement: Supplementary file 1 [file Table1.docx]

*Supplementary Table 1: Search terms in Pubmed*

**Supplementary table 1:**

**Search terms in Pubmed**

| **First term (" ")** | **AND** | **Second term (" ")** |
| --- | --- | --- |
| Combination vaccine |  | Alphavirus |
| Combined |  | Anthrax |
| Multi-pathogen vaccine |  | Arenavirus |
| Multipathogen vaccine |  | Bacillus anthracis |
| Multivalent |  | Botulinum |
| Bivalent |  | Botulism |
| Trivalent |  | Brucella |
| Tetravalent |  | Burkholderia |
|  |  | Clostridium |
|  |  | Coxiella burnetii |
|  |  | eastern equine |
|  |  | Ebola |
|  |  | Filovirus |
|  |  | Flavivirus |
|  |  | Francisella |
|  |  | hantavirus |
|  |  | Lassa |
|  |  | Marburg |
|  |  | Nipah virus |
|  |  | Poxvirus |
|  |  | Q fever |
|  |  | Vaccinia |
|  |  | Venezuelan equine |
|  |  | western equine |
|  |  | Yersinia |
